# Structure‐Guided Modulation of the Catalytic Properties of [2Fe−2S]‐Dependent Dehydratases

**DOI:** 10.1002/cbic.202200088

**Published:** 2022-03-23

**Authors:** Okke Melse, Samuel Sutiono, Magdalena Haslbeck, Gerhard Schenk, Iris Antes, Volker Sieber

**Affiliations:** ^1^ Chair of Chemistry of Biogenic Resources Campus Straubing for Biotechnology and Sustainability Technical University of Munich Schulgasse 16 94315 Straubing Germany; ^2^ SynBiofoundry@TUM Technical University of Munich Schulgasse 22 94315 Straubing Germany; ^3^ TUM Center for Functional Protein Assemblies Technical University of Munich Ernst-Otto-Fischer-Straße 8 85748 Garching Germany; ^4^ School of Chemistry and Molecular Biosciences The University of Queensland 68 Cooper Road 4072 St. Lucia Australia; ^5^ Sustainable Minerals Institute The University of Queensland 47 Staff House Road 4072 St. Lucia Australia; ^6^ Catalytic Research Center Technical University of Munich Ernst-Otto-Fischer-Straße 1 85748 Garching Germany

**Keywords:** biocatalysts, bioinformatics, dehydratases, enzyme catalysis, structure-activity relationships

## Abstract

The FeS cluster‐dependent dihydroxyacid dehydratases (DHADs) and sugar acid‐specific dehydratases (DHTs) from the ilvD/EDD superfamily are key enzymes in the bioproduction of a wide variety of chemicals. We analyzed [2Fe−2S]‐dependent dehydratases *in silico* and *in vitro*, deduced functionally relevant sequence, structure, and activity relationships within the ilvD/EDD superfamily, and we propose a new classification based on their evolutionary relationships and substrate profiles. *In silico* simulations and analyses identified several key positions for specificity, which were experimentally investigated with site‐directed and saturation mutagenesis. We thus increased the promiscuity of DHAD from *Fontimonas thermophila* (*Ft*DHAD), showing >10‐fold improved activity toward D‐gluconate, and shifted the substrate preference of DHT from *Paralcaligenes ureilyticus* (*Pu*DHT) toward shorter sugar acids (recording a six‐fold improved activity toward the non‐natural substrate D‐glycerate). The successful elucidation of the role of important active site residues of the ilvD/EDD superfamily will further guide developments of this important biocatalyst for industrial applications.

## Graphical abstract

**Enzyme catalysis**: *In silico* and *in vitro* investigations enabled the elucidation of the role of important active site residues of the enzymes dihydroxyacid dehydratase (DHAD) and sugar acid‐specific dehydratase (DHT), both belonging to the FeS cluster‐dependent ilvD/EDD superfamily. We proposed a new classification for these enzymes, and we were able to alter their substrate preference, improving activities for non‐natural substrates significantly.

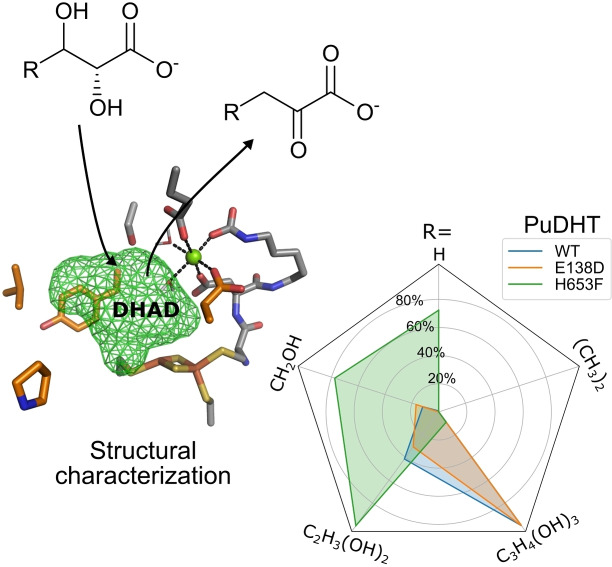

## Introduction

**Scheme 1:**
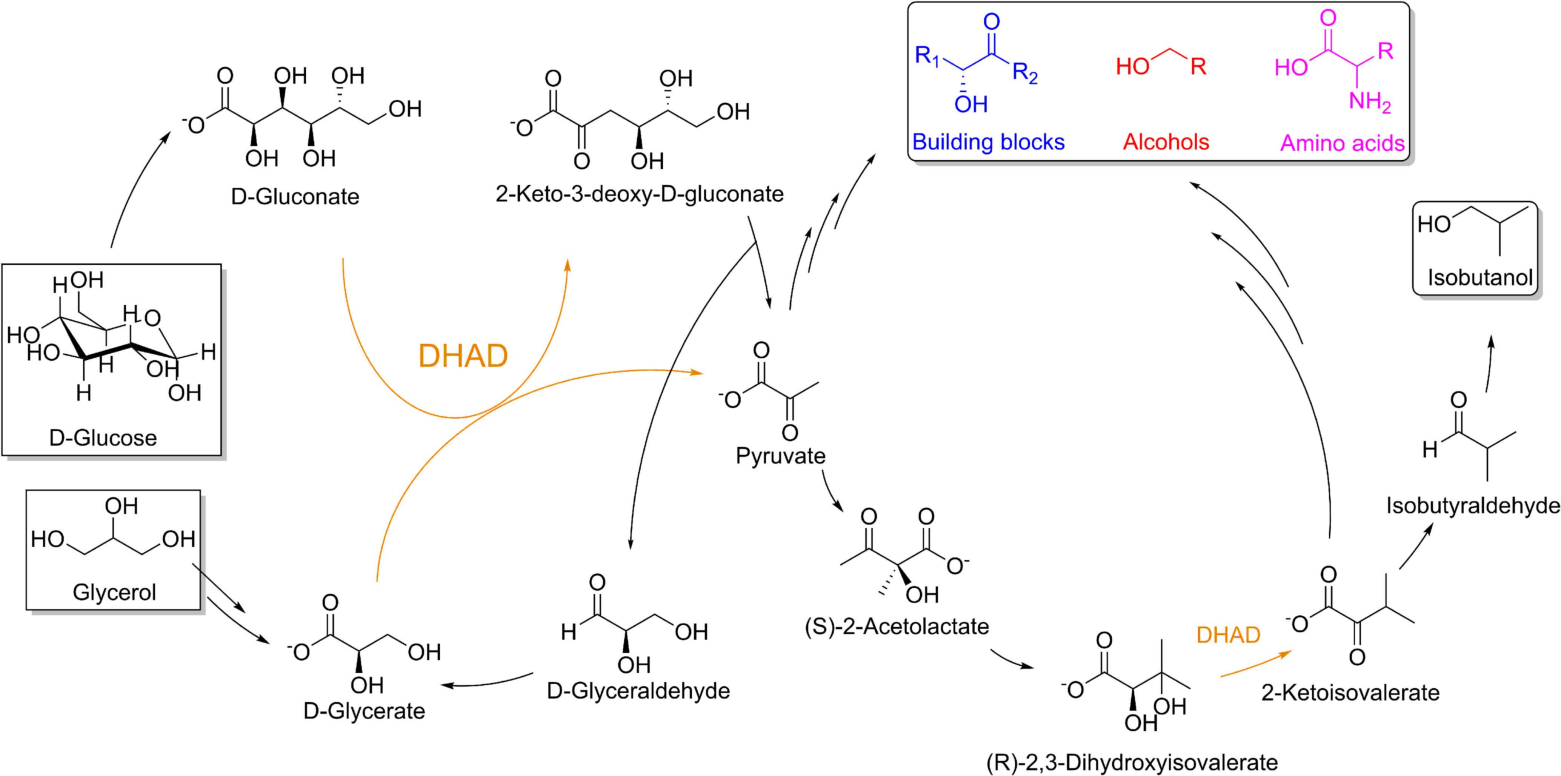
Minimized synthetic pathway for the production of isobutanol from D‐glucose and glycerol enabled by *Ss*DHAD (orange). Furthermore, the dehydration products of the *Ss*DHAD‐catalyzed reaction, *i. e*. pyruvate and 2‐ketoisovalerate, can be converted into additional chemical building blocks, alcohols, and amino acids (shown in blue, red and magenta, respectively).

Production of higher chain alcohols, such as isobutanol, has gained major interest in the last decade because of their potential as biofuels with properties similar to gasoline. Isobutanol has a higher energy density and is less hygroscopic than ethanol, the traditional biofuel.^[1]^ Bioproduction of isobutanol from fermentation can be achieved by a modified Ehrlich pathway, *i. e*. catabolism of branched chain amino acids (BCAA).^[2]^ One key enzyme in this pathway is dihydroxyacid dehydratase (DHAD; Scheme 1), which catalyzes the dehydration of (*R*)‐2,3‐dihydroxyisovalerate (DHIV) to 2‐ketoisovalerate (KIV). As an alternative to the fermentation approach, a cell‐free system with minimized cofactor and enzyme requirements was developed.^[3]^ This *in vitro* system is able to convert D‐glucose to isobutanol utilizing only eight enzymes, in contrast to the 15 enzymes needed in the *in vivo* approach. The key enzyme in this system is a promiscuous DHAD from *Saccharolobus solfataricus* (*Ss*DHAD). In addition to the dehydration of DHIV to KIV, this enzyme also catalyzes the dehydration of the sugar acids D‐gluconate to 2‐keto‐3‐deoxy‐D‐gluconate (KDG) and D‐glycerate to pyruvate (Scheme 1).^[4]^ While the first reaction is part of the semi‐ or non‐phosphorylative Entner‐Doudoroff (ED) pathway, the latter is a non‐natural reaction that proceeds at a very slow rate, thus serving as the major bottleneck in the cell‐free system.[^3^, ^5^] The dehydration of D‐glycerate is also the key step in the valorization of glycerol, thus enhancing the significance of DHAD for applications in biotechnology.[^6^, ^7^]

In contrast to DHADs, which use DHIV as preferred substrate, a closely related group of dehydratases (DHTs) prefer various sugar acids. D‐xylonate DHT from *Caulobacter crescentus* (*Cc*XylDHT) and L‐arabinonate DHT from *Rhizobium leguminosarum* (*Rl*ArDHT) have recently been characterized; both are candidate alternatives for *Ss*DHAD in the *in vitro* cascade.^[8]^ However, while *Cc*XylDHT and *Rl*ArDHT are reactive toward long sugar acid substrates (*e. g*. D‐gluconate) we recently showed that they are practically inactive toward D‐glycerate.^[9]^ In that study, several novel dehydratases (DHADs and DHTs) with activity toward D‐glycerate were described, with the DHAD from *Fontimonas thermophila* (*Ft*DHAD) and the DHT from *Paralcaligenes ureilyticus* (*Pu*DHT) being the most promising. Both showed up to 50‐fold higher activity toward D‐glycerate when compared to *Ss*DHAD. Further substrate profiling demonstrated however that *Ft*DHAD, in contrast to *Cc*XylDHT and *Rl*ArDHT, is significantly less active toward longer sugar acids (such as D‐gluconate), while *Pu*DHT is not active on branched‐chain substrates such as DHIV, in contrast to *Ss*DHAD (Table 1).^[9]^

**Table 1:** Experimental specific activities of DHTs and DHADs toward sugar acid substrates of varying size and the branched chain acid DHIV.

	V (U/mg)^[e]^				
	D‐glycerate	L‐threonate	D‐xylonate	D‐gluconate	DHIV
*Pu*DHT^[a]^	0.31±0.03	5.71±0.98	19.86±1.06	48.23±1.18	n. a.
*Rl*ArDHT^[b]^	<0.01±0.00	n. d.	6.19±0.76	7.65±0.71	n. d.
*Cc*XylDHT^[b]^	<0.01±0.00	n. d.	25.65±0.67	47.91±2.86	n. d.
*Ft*DHAD^[c]^	0.96±0.07	2.23±0.17	0.36±0.01	n. a.	12.92±0.63
*Mt*DHAD^[12]^	n. d.	n. d.	n. d.	n. d.	1.87±0.06
*Ath*DHAD^[17]^	n. d.	n. d.	n. d.	n. d.	Active^[d]^
*Ss*DHAD^[c]^	0.02±0.00	0.03±0.00	2.18±0.03	0.77±0.04	0.75±0.03

[a] Activity determined in this study at 30 °C. [b] Activity toward D‐glycerate was taken from Sutiono *et al*. (2020)^[9]^ and that toward D‐xylonate and D‐gluconate from Andberg *et al*. (2016).^[8]^ [c] Activities in this study were determined at 50 °C. The loading of [Fe−S] was not determined. Thus, although the values of the activities for the DHADs may be higher than reported here, the substrate preference will remain the same. [d] Activity was determined, but no absolute value provided in cited study. [e] Activity of *Pu*DHT, *Ft*DHAD and *Ss*DHAD was determined with a substrate concentration of 25 mM. n. d.=activity was not determined in the previous studies; n. a.=activity <0.01 U/mg.

**Figure 1:**
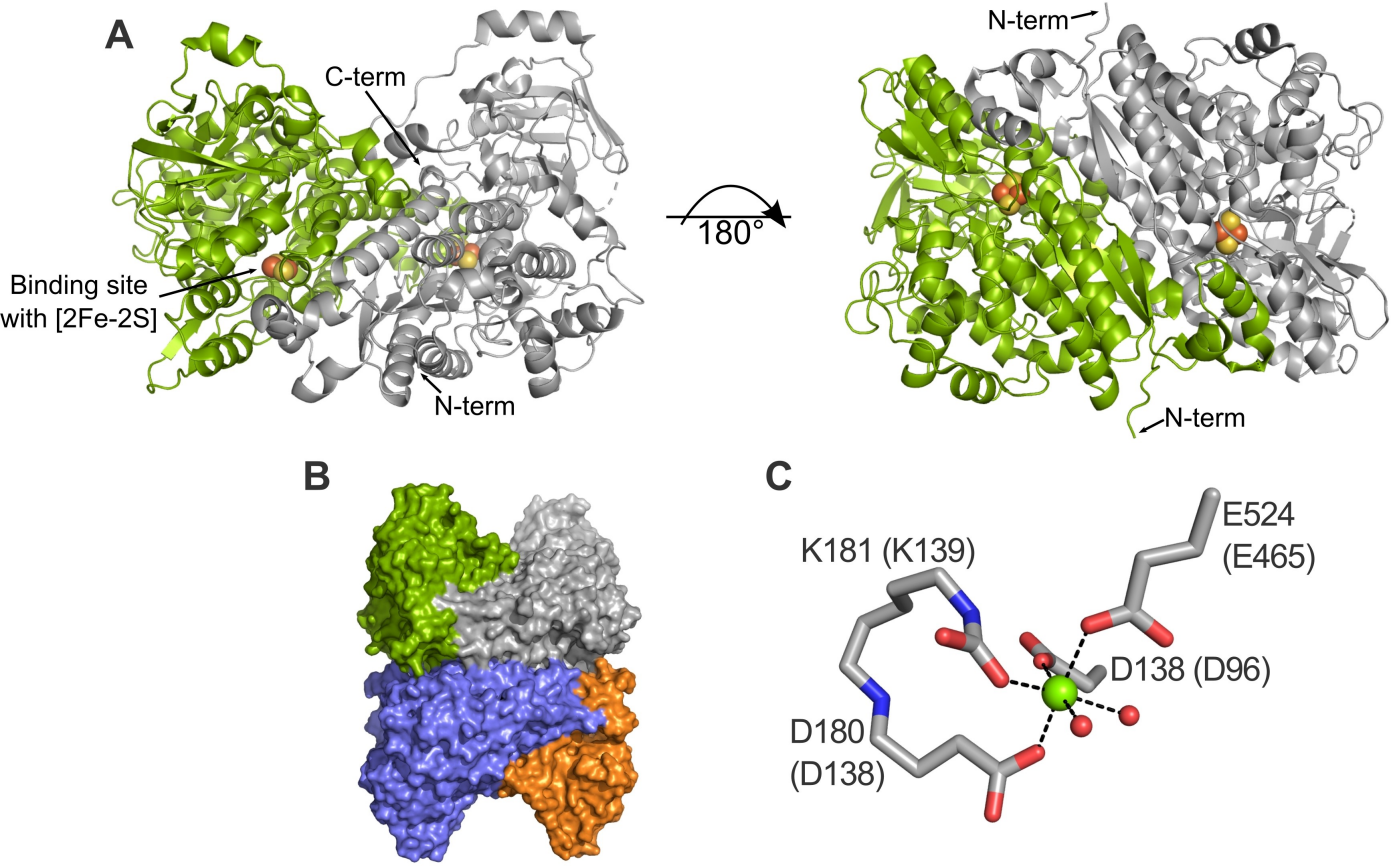
Oligomerization of [2Fe−2S]‐dependent dehydratases. The figures were created based on a DHAD from *M. tuberculosis* (*Mt*DHAD, PDB: 6ovt). The single monomers are shown as respective green and grey cartoon, forming a dimer (A). The termini and the binding site are indicated in the figure. Two dimers pack together, forming a tetramer (B). The octahedral coordination of the Mg^2+^ ion is completed by water molecules in the X‐ray structure (C). The alignment numbers (see Figure S1) are used for residue numbering; residue numbers from the original sequence of *Mt*DHAD are shown in brackets.

DHTs and DHADs belong to the ilvD/EDD (**i**soleucine, **l**eucine, **v**aline **D**ehydratase/**E**ntner‐**D**oudoroff **D**ehydratase) superfamily of enzymes.[^4^, ^8^, ^10^] To date, there is no consistent naming system for enzymes from this superfamily, which complicates their comparative analysis. Most of the characterized representatives are tetrameric, containing four identical monomers arranged as a dimer of homodimers. Each dimer contains two active sites, located at the dimer interface (Figure 1). Despite the central role of these enzymes in metabolism and potential in biotechnology, X‐ray structures are currently only available for a few of these enzymes including the DHADs from *Arabidopsis thaliana* (*Ath*DHAD; PDB: 5ze4)^[11]^ and *Mycobacterium tuberculosis* (*Mt*DHAD; PDB: 6ovt),^[12]^ and the DHTs *Cc*XylDHT (PDB: 5oyn)^[13]^ and *Rl*ArDHT (PDB: 5j84/5j85).^[14]^ This paucity of available structures limits both in‐depth comparative functional studies among members of the ilvD/EDD superfamily and the (semi‐)rational engineering of variants with properties suitable for industrial applications. No *in silico* studies of DHADs or DHTs belonging to the ilvD/EDD superfamily have yet been reported, partially due to the limited availability of experimental structures, but also because of the complex nature of the catalytic site, which requires both an FeS cluster ([2Fe−2S] or [4Fe−4S]) and a nearby Mg^2+^ ion (Figure 1).[^13^, ^14^] The complex electronic properties of the FeS cluster and the highly polarized binding site are difficult to parameterize and simulate, making computational studies challenging and time‐consuming. There are also no bound substrates in any of the available DHAD or DHT X‐ray structures. Therefore, molecular docking simulations are required to identify likely binding poses of the substrates, as well as residues that play an important role in substrate binding and catalysis. Such residues may represent mutational hotspots to engineer DHAD/DHT variants with tailored properties.

In this work, we introduce a new classification system, which will allow more straightforward comparison among members of the ilvD/EDD superfamily based on their substrate profiles and evolutionary relationship, in particular the [2Fe−2S]‐dependent dehydratases. Furthermore, we used the available crystal structures of DHADs and DHTs to build homology models of *Ss*DHAD, *Ft*DHAD and *Pu*DHT. These models, together with computational simulations, allowed predictions of the origin of the substrate selectivity of these enzymes. We used this insight to rationally design variants of these three enzymes with improved activity toward substrates of interest. The success of our predictions was highlighted in the H653F variant of *Pu*DHT (residue numbering according to the sequence alignment; Figure S1), which has a six‐fold higher activity toward D‐glycerate than its wild‐type counterpart. This study sheds light on the sequence, structure and activity relationship within the ilvD/EDD superfamily and thus facilitates targeted bioengineering studies to design optimized biocatalysts for industrially relevant biotransformations.

## Results and Discussion

### Substrate profiles and phylogenetic relationship: New classification of DHADs and DHTs

In previous work we identified dehydratases that are active on D‐glycerate and partially characterized more than 20 enzymes.^[9]^ We focused primarily on the dehydratases that contain a [2Fe−2S] center because of their relative stability in the presence of oxygen.^[15]^ These enzymes can be grouped into two clusters based on their substrate profiles. The first cluster consists of DHTs, which are active toward longer sugar acids, and the second cluster contains DHADs, which have a preference for branched dihydroxyacids such as DHIV. We used one enzyme from each cluster as model enzyme in this work, namely *Pu*DHT and *Ft*DHAD.^[9]^ We included *Ss*DHAD in our study as this enzyme was reported to be active on both substrate classes.^[4]^ We characterized these enzymes under similar experimental conditions, and compared the results to other known dehydratases (Table 1). In the assays, sugar acids with increasing chain length (*i. e*. from D‐glycerate *via* L‐threonate and D‐xylonate to D‐gluconate) and the branched DHIV were used as substrates. The concentration for each substrate was 25 mM, which is well‐above the K_M_ of previously characterized DHADs and DHTs.[^4^, ^8^, ^9^, ^12^] Furthermore, we focused primarily on relative activities rather than absolute values in order to compare the change in the substrate profiles of the investigated enzymes.

**Figure 2:**
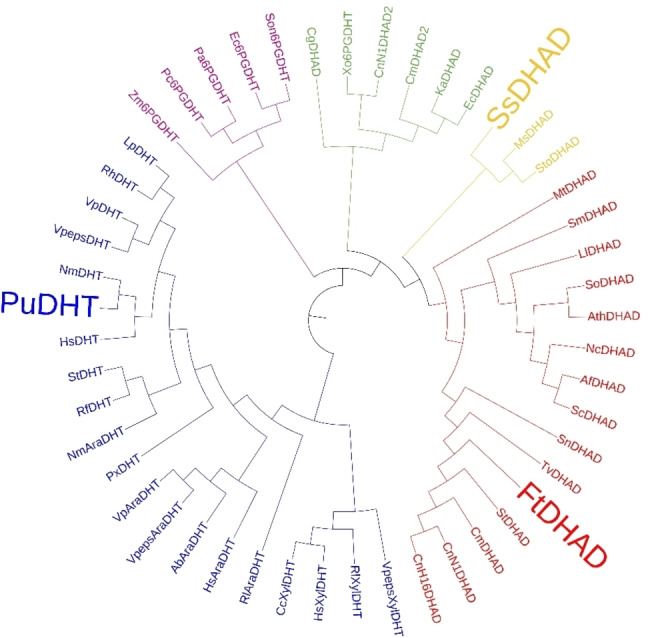
Phylogenetic tree of characterized and selected putative enzymes from the IlvD/EDD superfamily. The blue branch represents sugar acid dehydratases (SADHTs) and the red branch represents DHADs active on branched chain acids (BCADHTs). The yellow branch represents promiscuous DHADs active on sugar acids and branched chain acids (PADHT). *Sto* and *Ms*DHADs have not yet been characterized but were included in the tree to highlight the PADHT clade. The green branch represents DHADs, which contain a [4Fe‐4S] cluster, and the purple branch represents 6‐phosphogluconate dehydratases. The tree was constructed using MAFFT with the default method and visualized using iTOL.[^20^, ^21^]

In agreement with our previous study, *Pu*DHT is most active toward D‐gluconate, with the activity decreasing as the chain length of the sugar acid substrate decreases; no activity toward DHIV is recorded (Table 1). In contrast, *Ft*DHAD has virtually no activity toward D‐gluconate, but is active toward shorter sugar acid substrates, in particular L‐threonate. Maximum activity for *Ft*DHAD is recorded with its biological substrate, DHIV, more than five‐times faster than that measured with L‐threonate. The substrate preference of *Ss*DHAD lies somewhere in between those of *Pu*DHT and *Ft*DHAD with the relatively large sugar acid D‐xylonate being the most preferred reactant, but the enzyme displaying significant activity for DHIV as well.^[16]^ To explore the evolutionary relationship of these dehydratases further, we constructed a phylogenetic tree based on the sequences of all reported DHADs and DHTs in the literature (Figure 2). Both *Ft*DHAD and *Ss*DHAD cluster with other DHADs such as the enzymes from *A. thaliana* (*Ath*DHAD) and *M. tuberculosis* (*Mt*DHAD). However, *Ss*DHAD appears to have diverged from other DHADs early, maintaining larger similarities to DHTs. *Pu*DHT clusters closely with other known DHTs, including *Cc*XylDHT and *Rl*ArDHT.

Although DHADs and DHTs all belong to the ilvD/EDD superfamily, only DHADs are associated with a function in BCAA biosynthesis (due to their activity toward DHIV). DHTs, with preference for sugar acids, are key enzymes in the oxidative pentose (OP) pathways (Dahms and Weimberg pathways).[^18^, ^19^] *Ss*DHAD, on the other hand, appears to occupy a unique evolutionary position, demonstrating significant activity toward both sugar acids and DHIV, and is thus believed to play a role in the BCAA, ED and OP pathways. However, strictly speaking, since all of these substrates contain a hydroxyl group at the α‐ (C2) and β‐positions (C3) of an acid substrate, all of these enzymes are, in fact, dihydroxyacid dehydratases, *i. e*. DHADs. Thus, to make a clearer distinction, we propose another naming scheme, where enzyme homologous to *Pu*DHT, *Cc*XylDHT and *Rl*ArDHT are named **s**ugar **a**cid **d**e**h**ydra**t**ases (SADHTs), and enzymes homologous to *Ft*DHAD, which are predominantly active toward DHIV, are named **b**ranched **c**hain **a**cid **d**e**h**ydra**t**ases (BCADHTs). Finally, enzymes that display comparable activities toward sugar and branched chain acid substrates (such as *Ss*DHAD) are labelled as **p**romiscuous **a**cid **d**e**h**ydra**t**ases (PADHTs). To date, *Ss*DHAD is the only PADHT that has been characterized but the phylogenetic inference (Figure 2) suggests that enzymes such as *Sto*DHAD from *Sulfurisphaera tokodaii* and *Ms*DHAD from *Metallosphaera sedula* should also have a promiscuous substrate profile.

### *In silico* modeling of representative DHADs

To gain structural insights relevant to the engineering of DHADs with optimized catalytic properties, homology models of representative DHADs for each of the three classes were generated (*i. e. Pu*DHT, *Ft*DHAD and *Ss*DHAD). The crystal structures of *Ath*DHAD, *Mt*DHAD, *Cc*XylDHT and *Rl*ArDHT were used as templates. A multiple sequence comparison was performed to align all templates and target proteins (Figure S1). Since residues sharing an alignment position are located in the same three‐dimensional space in the protein, the alignment positions allow easier identification and comparison of residues based on their location in the protein. Therefore, we used the alignment positions of amino acids, rather than their position in the sequence throughout the remainder of this study. The correlation between sequence and alignment positions for relevant residues is shown in Table S1. The homology models (Figure S2) scored well during the QMEAN^[22]^ quality estimation analysis, with QMEAN4 Z‐scores ranging from −1.34 to −0.62 (Table S2), indicating a high quality for the homology models (see the Computational Biology section in Supporting Information for further details).

**Figure 3:**
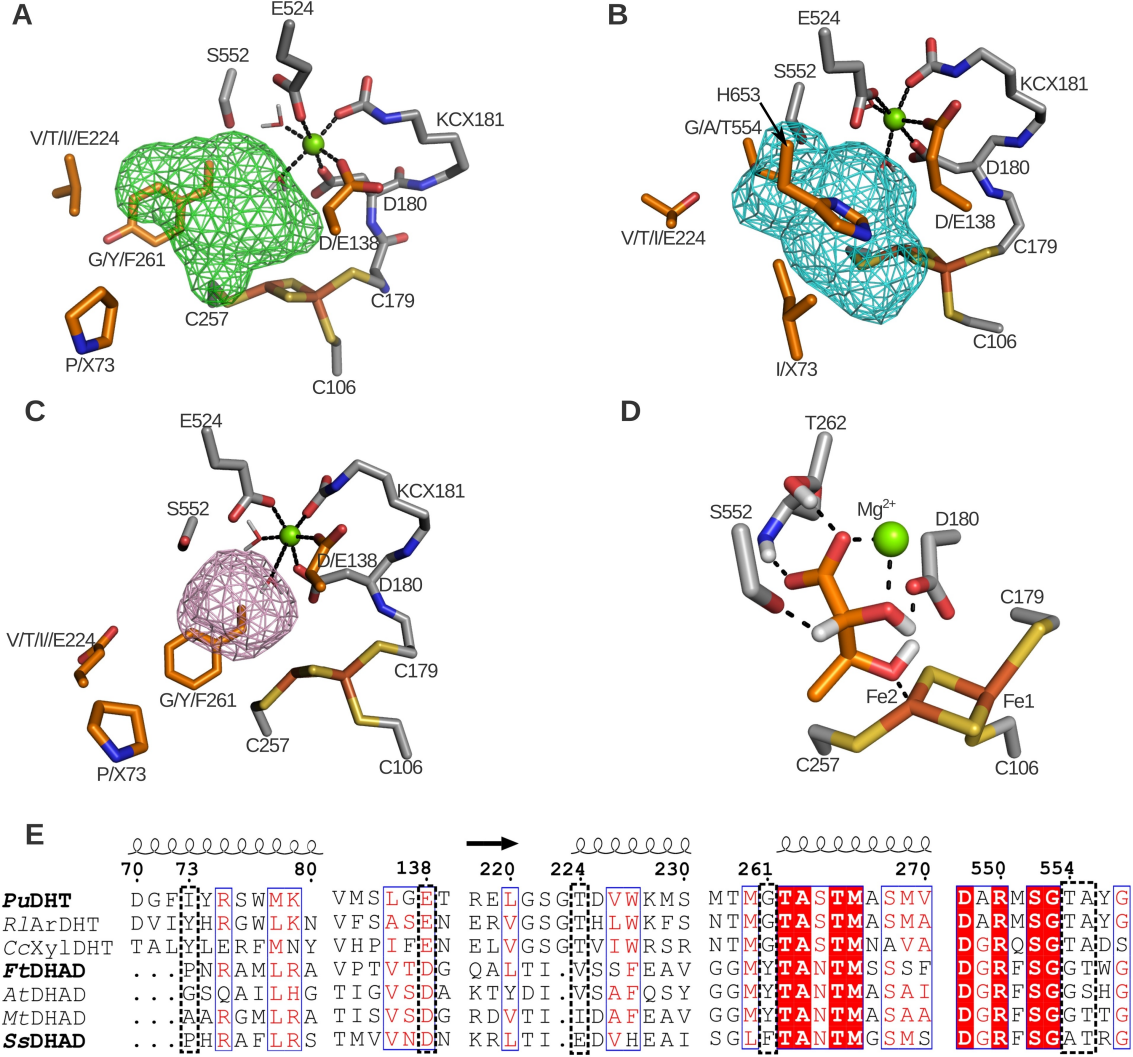
Structural representation of the binding site of *Ft*DHAD (A), *Pu*DHT (B) and *Ss*DHAD (C). The binding pocket is visualized by the mesh surface and residues identified as mutation hotspots and other relevant binding site residues are shown as orange and grey sticks, respectively. DHIV docked in *Ft*DHAD is used to visualize the conserved binding mode of dihydroxyacids in DHADs (D). Alignment numbers are used for the residue numbering, and amino acid side chains are indicated using their single letter abbreviation, except KCX, which stands for carboxylated lysine; a singular X indicates any amino acid. Note that the amino acid at alignment position 73 originates from the other monomer. The sequence alignment of the hotspot regions is shown (E), where the numbers above the sequences indicate the alignment numbers, and helices and arrows indicate the secondary structure of that corresponding sequence (alpha‐helix or beta‐sheet, respectively). The dotted boxes highlight the mutation hotspots.

Two models for the reaction mechanism employed by DHADs and DHTs were recently proposed. Rahman *et al*.,^[14]^ based on their studies with *Cc*XylDHT, suggested a mechanism for the dehydration reaction where the proton from the C2 atom of the substrate (Scheme 1) is removed by the deprotonated alkoxide side chain of the serine residue in alignment position 552 (see Figure 3 for details). The resulting carbanion is stabilized by the Mg^2+^ in the active site, leading to a weakening of the C3−O bond. Subsequently, one of the iron atoms of the [2Fe−2S] cluster (Fe2) promotes the abstraction of the hydroxyl group from C3, which triggers the tautomerization of the product to its keto form. This mechanistic model is supported by mutagenesis data that demonstrate the essential role of Ser552, as well as Mössbauer and EPR spectra that highlight the significance of the Lewis acid behavior of the [2Fe−2S] cluster in the catalytic cycle.[^11^, ^12^, ^13^] More recently, an alternative reaction mechanism was proposed for the DHIV dehydration catalyzed by a DHAD from the cyanobacterium *Synechocystis* sp. PCC 6803 (*Sn*DHAD).^[23]^ In this model, the C2 hydrogen is abstracted by a base (possibly a water molecule coordinated by Asp180). Subsequently, Ser552 donates a proton to 3‐OH and thereby facilitates elimination of this hydroxyl group, releasing a water molecule. We performed docking simulations (see below) of relevant substrates in the homology models of *Ss*DHAD, *Ft*DHAD and *Pu*DHT, which largely support the reaction mechanism proposed by Rahman and colleagues. All of our docking simulations indicate that Ser552 is in close proximity to the C2 hydrogen, Fe2 is near C3−OH and the Mg^2+^ ion is close to C2−OH, where it can stabilize the negative charge of the carbanion (Figure 3).

### Hotspot identification

From the multiple sequence alignment (Figure S1), a number of conserved residues were identified within each of the three DHT classes (*i. e*. SADHT, BCADHT and PADHT), including the three cysteine residues that coordinate the two iron atoms of the FeS cluster (Cys106, Cys179 and Cys257), and the catalytic serine residue (Ser552), but there are some interesting variations. For example, the motif around the second cysteine ligand of the [2Fe−2S] cluster (Cys179; underlined) is identical for SA‐ and PADHTs (*i. e*. GCDKTT; Figure S1) but is replaced by GCDKNM in BCADHTs. However, in the motif surrounding the third cysteine ligand of the FeS cluster (Cys257), PA‐ and BCADHTs are well conserved (GTCSG *vs* GACGG), whereas the SADHTs are less so (GHCM/NT). Since representatives from the three DHT classes differ significantly in their substrate profiles while maintaining a similar overall structure and mechanism (Table 1), residues with high intra‐class but low inter‐class conservation are expected to be major determining factors (*i. e*. “hotspots”) that control substrate specificity in these enzymes. Using the homology models of *Pu*DHT, *Ft*DHAD and *Ss*DHAD (Figure S2), an amino acid sequence comparison (Figure S1) and *in silico* substrate docking (Figure 3D), we identified several hotspots. Only residues in or within proximity of the active site were considered. The identified hotspots are (alignment positions): 73, 138, 224, 261, 554–555 (SGXX motif) and the C‐terminal histidine of SADHTs (Figure 3A–C, E). We performed site‐directed mutagenesis or saturation mutagenesis of these hotspots to validate our predictions, and to define the contribution of each of these hotspots on the substrate profile and enzyme activity (Table 2).

**Table 2:** Relative activity of *Pu*DHT, *Ft*DHAD and *Ss*DHAD variants against substrates with varying size. The highest activity observed in the wild‐type enzyme was set as 100 %. The sequence numbers are the alignment positions.^[a]^

	D‐glycerate	L‐threonate	D‐xylonate	D‐gluconate	DHIV
*Pu*DHT – WT	0.65±0.06	11.90±2.04	41.38±2.20	100.00±2.45	n. a.
E138D	n. a.	0.71±0.06	1.23±0.03	4.23±0.42	n. a.
E138A	n. d.	n. d.	n. d.	0.05±0.00	n. d.
T224E	n. a.	n. a.	n. a.	n. a.	n. a.
G261F	n. a.	n. a.	n. a.	n. a.	n. a.
G261Y	n. a.	n. a.	n. a.	n. a.	n. a.
T554G, A555T (SGGT)	n. a.	n. a.	n. a.	n. a.	n. a.
H653F (C‐terminus)	4.02±0.22	4.31±0.93	5.56±0.06	0.50±0.02	n. a.
*Ft*DHAD – WT	7.44±0.56	18.04±1.30	2.81±0.07	0.02±0.00	100.00±4.91
P73G	1.76±0.1	4.68±1.72	1.55±0.14	0.22±0.01	64.20±7.59
D138E	0.10±0.00	2.04±0.13	0.15±0.00	n. a.	16.55±1.15
D138A	n. d.	n. d.	n. d.	n. d.	0.04±0.00
V224E	0.02±0.01	0.14±0.01	0.03±0.01	n. a.	0.60±0.08
Y261G	0.03±0.00	0.32±0.00	0.11±0.00	n. a.	1.03±0.04
G554T, T555A (SGTA)	n. a.	0.08±0.00	n. a.	n. a.	n. a.
*Ss*DHAD – WT	1.12±0.00	1.58±0.09	100.00±1.54	35.5±1.78	34.73±1.16
D138E	0.92±0.00	1.28±0.02	11.53±1.50	16.76±2.30	22.54±3.34
D138A	n. d.	n. d.	0.23±0.01	n. d.	n. d.
A554T, T555A (SGTA)	n. a.	n. a.	n. a.	n. a.	n. a.

[a] n. d.=not determined; n. a.=relative activity <0.01 %.

### Influence of alignment position 73 on substrate specificity and catalytic efficiency

**Figure 4:**
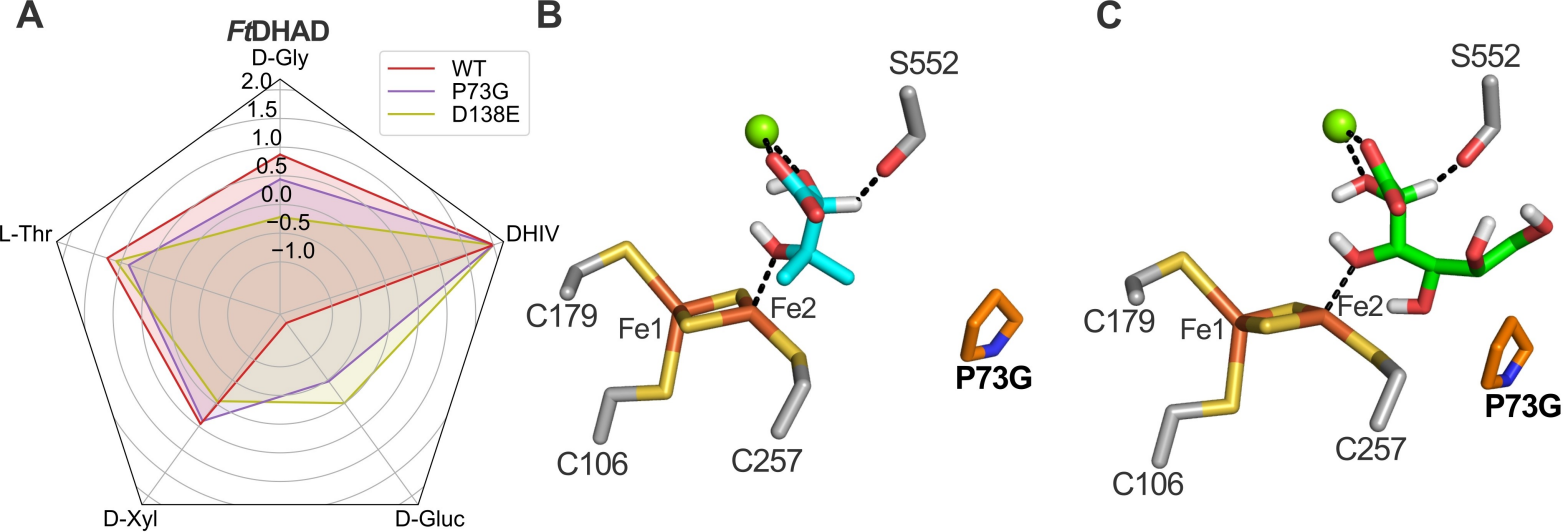
Effect of site‐directed mutations on the substrate profile of *Ft*DHAD. Relative activities of *Ft*DHAD variants shown in a radar plot in logarithmic scale, with the highest observed activity set to 100 % (A). Exact values are provided in Table 2. Docking of DHIV (B) and D‐gluconate (C) in the active site of *Ft*DHAD. P73 is shown as orange sticks. Alignment numbers are used for the residue numbering.

The residue located in alignment position 73 is on an N‐terminal helix of a monomer of the DHAD/DHT homodimer (Figure S2); however, this residue forms part of the active site of the other monomer (Figures 3 and S2). Diverse residues of varying size are found at this position, ranging from proline in *Ss*DHAD and *Ft*DHAD, to the polar and bulky tyrosine in *Rl*ArDHT and *Cc*XyDHT (Figure 3). In *Pu*DHT an isoleucine is found in this position, and saturation mutagenesis did not result in variants with a significant change in specificity toward D‐gluconate, L‐threonate and DHIV (Figure S3). This observation stands in contrast to an earlier study on the structure of *Cc*XylDHT, which predicted that the residue in this position is important for the substrate preference of SADHTs.^[13]^ The activity landscape of the *Ft*DHAD P73X library toward DHIV indicates that >70 % of the variants show a significant decrease in activity in comparison to the wild‐type enzyme (Figure S3). Decreased activity was also observed toward L‐threonate. However, several variants in this library were observed that have almost >three‐fold higher activity toward D‐gluconate than wild‐type *Ft*DHAD. Importantly, and in contrast to the wild‐type enzyme, three of them display comparable activity toward DHIV and L‐threonate (Figure S3). Since our main goal is to find enzymes which show enhanced substrate promiscuity, we selected one of these three variants for further studies. It contains the proline to glycine substitution at position 73, and has a >10‐fold higher activity toward D‐gluconate, while retaining ∼64 % of the activity toward DHIV when compared to wild‐type *Ft*DHAD (Table 2 and Figure 4A). A possible explanation for this observed effect is a slight increase of binding site volume. Thus, the combined data demonstrate that the amino acid at position 73 plays an important role in altering the substrate preference of a BCADHT to that of a SADHT, but not *vice versa* (*i. e. Pu*DHT is not sensitive toward mutations in that position).

### Influence of alignment position 138 on Mg^2+^ coordination

Apart from the FeS cluster, members of the ilvD/EDD superfamily also require a Mg^2+^ ion (or other divalent cations) in the active site for their catalytic function. The majority of the residues coordinating this ion are highly conserved and include D180 and E524, as well as a carboxylated lysine (KCX181) and two water molecules (Figure 1C). Some modest variability is observed with the ligand in alignment position 138, where an aspartate is present in BCADHTs but a glutamate in SA‐ and PADHTs (Figure 3E). Our docking studies reveal that sugar acid substrates replace the water molecules and coordinate the Mg^2+^ ion with one carboxyl oxygen and the C2−OH, thereby completing the octahedral coordination sphere of the metal ion (Figure 3D). To evaluate the effect of the non‐conserved residue in position 138 on the catalytic properties of the dehydratases, we performed the following mutations: E138D and E138A in *Pu*DHT and D138E and D138A in *Ft*DHAD and *Ss*DHAD (Table 2). In all variants containing the alanine substitution a virtually complete loss of activity was observed for all substrates, likely to be due to impaired Mg^2+^ binding. The E138D mutation in *Pu*DHT had a similar effect, possibly because the shorter aspartate side chain is not able to interact effectively with the Mg^2+^. Less dramatic is the effect of the D138E substitution in *Ft*DHAD and *Ss*DHAD, but in general a reduction of the activity was observed for each substrate tested. The *in silico* simulations could not provide a conclusive explanation for this effect, but the longer side chain of glutamate may lead to a shift of the Mg^2+^ ion away from the [2Fe−2S] cluster, and thereby prevent optimal substrate orientation. Importantly, while the residue in position 138 clearly plays an essential role in Mg^2+^ binding, none of the mutations in this position had a significant effect on the substrate specificity of the dehydratases.

### The effect of a negative charge in position 224 in the active site

The residues in alignment position 224 are either a hydrophobic branched chain amino acid in BCADHTs, a hydrophilic threonine in SADHTs or a negatively charged glutamate in the PADHT *Ss*DHAD (Figure 3). The promiscuity of *Ss*DHAD is beneficial for the cell‐free bioproduction of isobutanol (Scheme 1), a trait we aimed to establish in SADHTs and BCADHTs. We hypothesized that the negative charge at position 224 plays an important role in the promiscuity of PADHTs. Accordingly, T224E and V224E variants of *PuDHT* and *Ft*DHAD, respectively, were generated. This mutation, however, completely abolished the activity of *Pu*DHT toward all substrates tested (Table 2), and a similar effect was observed for *Ft*DHAD, although the mutant retained modest (<1 %) activity for most of the substrates. Thus, substituting an amino acid at this position in *Pu*DHT and *Ft*DHAD into a negatively charged glutamate is not sufficient to introduce the promiscuity of *Ss*DHAD into SADHTs or BCADHTs.

### Binding site volume (alignment position 261)

Another marked difference between the three classes of DHTs is observed in position 261. While glycine is present in the SADHTs, this small side chain is replaced by the much bulkier tyrosine and phenylalanine in the BCADHTs and PADHTs, respectively. An analysis of the active sites of the homology models for *Pu*DHT, *Ft*DHAD and *Ss*DHAD (Figure 3) reveals that these residues point inside the active site of these enzymes. Consequently, the SADHTs have a considerably larger active site volume than the other DHTs. Hence, in order to test if the active site volume plays an important role in their substrate selection, G261F and G261Y mutations were introduced in *Pu*DHT and the Y261G in *Ft*DHAD (we did not perform the corresponding mutation in *Ss*DHAD, *i. e*. F261G, as our primary interest was in broadening the substrate scope of *Pu*DHT and *Ft*DHAD to a level similar to that of *Ss*DHAD). The substitutions Y261G in *Ft*DHAD resulted in drastic loss of activity, while the substitutions G261F and G261Y in *Pu*DHT both completely abolished the activity toward all substrates. Thus, changing the binding volume of *Ft*DHAD and *Pu*DHT by single substitution is not enough to alter their substrate preferences.

### The role of the SGXX motif on the catalytic properties (alignment positions 552–555)

The crystal structure of the SADHT *Cc*XylDHT highlighted the crucial role of the highly conserved serine residue in position 552 in the reaction mechanism of ilvD/EDD dehydratases (see above).^[13]^ It has been proposed that the deprotonated side chain of Ser552 initiates the catalytic cycle by abstracting a proton from the C2 atom of the substrate. Furthermore, structural information from another SADHT, *Rl*ArDHT, also ascribes an important role for the threonine at position 554 in the mechanism, stabilizing the catalytic serine during the reaction.^[14]^ In BCADHTs and PADHTs a glycine or alanine, respectively, is located in this position. However, in these enzymes a threonine is present in position 555 (SGGT and SGAT, respectively), whereas an alanine is present in SADHTs instead (SGTA). In order to assess the importance of the residues in these positions for the mechanism of these ilvD/EDD dehydratases, a series of mutants were generated (Tables 2 and S3). For *Pu*DHT, the threonine in position 554 is indeed crucial for the activity toward its preferred substrate, D‐gluconate, with (>0.5 % remaining upon substitution with glycine or valine). Interestingly, the alanine in position 555 also plays an important role; upon its replacement by another threonine only ∼0.7 % of the activity remains. Furthermore, replacing the wild‐type Thr554‐Ala555 sequence in *Pu*DHT with the Gly554‐Thr555 sequence present in BCADHTs leads to a completely inactive double mutant (irrespective of the substrate used). A similar trend was observed for *Ft*DHAD, where the conversion of its native SGGT motif to the SGTA motif present in SADHTs led to the virtually complete inactivation of the enzyme (again irrespective of the substrates used; Table 2). The threonine in this motif is also important for the function of *Ft*DHAD, but contrasting the observations made for *Pu*DHT, its replacement by a non‐polar amino acid does not completely abolish the catalytic function of the enzyme (the SGGA variant retains over 18 % of the activity). However, in both enzymes it is essential that a non‐polar side chain is located next to this threonine ‐ replacing it with another threonine is highly detrimental to the activities of these enzymes (Table S3). The same observations were also made for corresponding mutations in the PADHT *Ss*DHAD. Hence, while functionally essential, residues of the SGxx motif do not play an important role in determining the substrate preference of these enzymes. This interpretation is supported by *in silico* mutation studies, suggesting that a potential interaction between the substrate and Thr554 in *Pu*DHT may also exist in the G554T−T555A double mutant of *Ft*DHAD (*i. e*. a variant with the SGTA motif present in DHT). Since the residues of the SGxx motif are located on a rather flexible loop, we performed MD simulations with wild‐type enzymes and variant forms with inverted motifs to probe if the mutations alter the loop dynamics. However, no significant difference was observed (data not shown). It is thus likely that long‐range interactions, possibly mediated via an extensive hydrogen bond network, may play an important role in the mechanism. An in‐depth analysis of such effects is beyond the scope of the current study.

### Altering the substrate specificity of DHADs toward D‐glycerate

**Figure 5:**
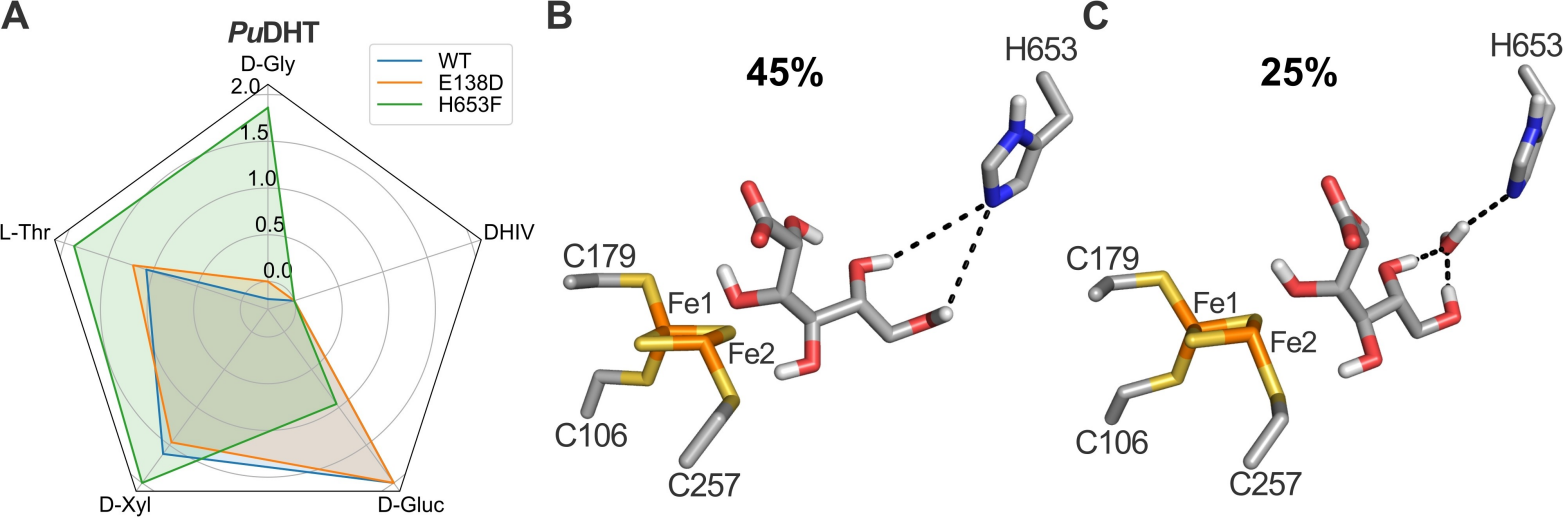
Effect of site‐directed mutations on the substrate profile of *Pu*DHT. Relative activities of *Pu*DHT variants shown in a radar plot in logarithmic scale with the highest observed activity set to 100 % (A). Exact values are provided in Table 2. Two preferred binding orientations for D‐gluconate in the active site of *Pu*DHT were modelled (B, C), where the percentage indicates the frequency of the MD simulations in which the respective binding mode was observed. Alignment numbers (Figure S1) are used for the residue numbering.

The hotspots identified in this study play an important role in the function of the ilvD/EDD dehydratases, and in some cases the substrate preference could be broadened through targeted mutations (*e. g*. the P73G mutation which widens the substrate preference of *Ft*DHAD to accept D‐gluconate; Table 2). However, none of the mutations in these hotspots altered the substrate preferences of the SA‐ and PADHTs. Furthermore, none of the variants displayed increased activity toward D‐glycerate, an important substrate in biomanufacturing processes (Scheme 1). The crystal structures of *Cc*XylDHT and *Rl*ArDHT and the homology model of *Pu*DHT reveal that the conserved C‐terminal histidine residue of the SADHTs extends toward the active site, located at the dimer interface. In contrast, in the available crystal structures of the BCADHTs *Ath*DHAD and *Mt*DHAD, as well as the homology models of *Ft*DHAD and *Ss*DHAD, the C‐terminal residue is not conserved and is not in the vicinity of the active site (Figures S1 and S4). Docking simulations were thus performed with the homology model of *Pu*DHT and various sugar acid substrates to probe if the C‐terminal histidine contributes to catalysis in SADHTs. We hypothesized that this residue may play a role in positioning sugar acid substrates in the active site, a hypothesis that is based on the observation that *Pu*DHT is more reactive toward larger sugar acids (Table 1). Indeed, the docking simulations demonstrate that both D‐gluconate and D‐xylonate are able to form a hydrogen bond with the C‐terminal histidine via their C5−OH groups; L‐threonate and D‐glycerate are too short to form a similar H‐bond (Figure S5). These findings were further supported by MD simulations with enzyme‐D‐gluconate and enzyme‐L‐threonate complexes. In these simulations, both C5−OH and C6−OH of D‐gluconate alternately form a hydrogen bond with the terminal histidine residue of *Pu*DHT (Figures 5 and S6), an interaction that is conserved throughout the entire MD simulation. In contrast, MD simulations with the docked enzyme‐L‐threonate complex confirmed that no interactions between this substrate and the C‐terminal histidine is formed. In order to substantiate these computational predictions, two saturation libraries for *Pu*DHT were generated. One library was designed to target the terminal histidine residue, and the other added an amino acid prior to this terminal histidine, thus extending the C‐terminal end by one amino acid. The two libraries were screened using different sugar acids and DHIV as substrates. The extension of the C‐terminal end showed a highly detrimental effect, completely inactivating the enzyme regardless of which substrate was used in the assays (Figure S7). Saturating the terminal histidine to other amino acids demonstrated a similar effect for the reaction with D‐gluconate. However, one variant (H653F) showed an improved activity toward D‐glycerate. While wild‐type *Pu*DHT is minimally active toward D‐glycerate (less than 1 % when compared to D‐gluconate), the variant is ∼six‐fold more reactive toward this substrate, and the ratio of D‐gluconate/D‐glycerate activity has improved >1000‐fold in comparison to the wild‐type enzyme (Table 2 and Figure 5A). Hence, the C‐terminal end plays indeed an important role in the mechanism of SADHTs. Extending the length of the C‐terminal end did not promote interactions with the smaller substrates, possibly because of steric clashes. However, replacing the native histidine by a slightly larger phenylalanine altered the size of the active site cavity sufficiently to exclude the large D‐gluconate substrate and favor binding of smaller sugar acids.

### Stereoselective substrate specificity of *Pu*DHT and *Ft*DHAD

**Figure 6:**
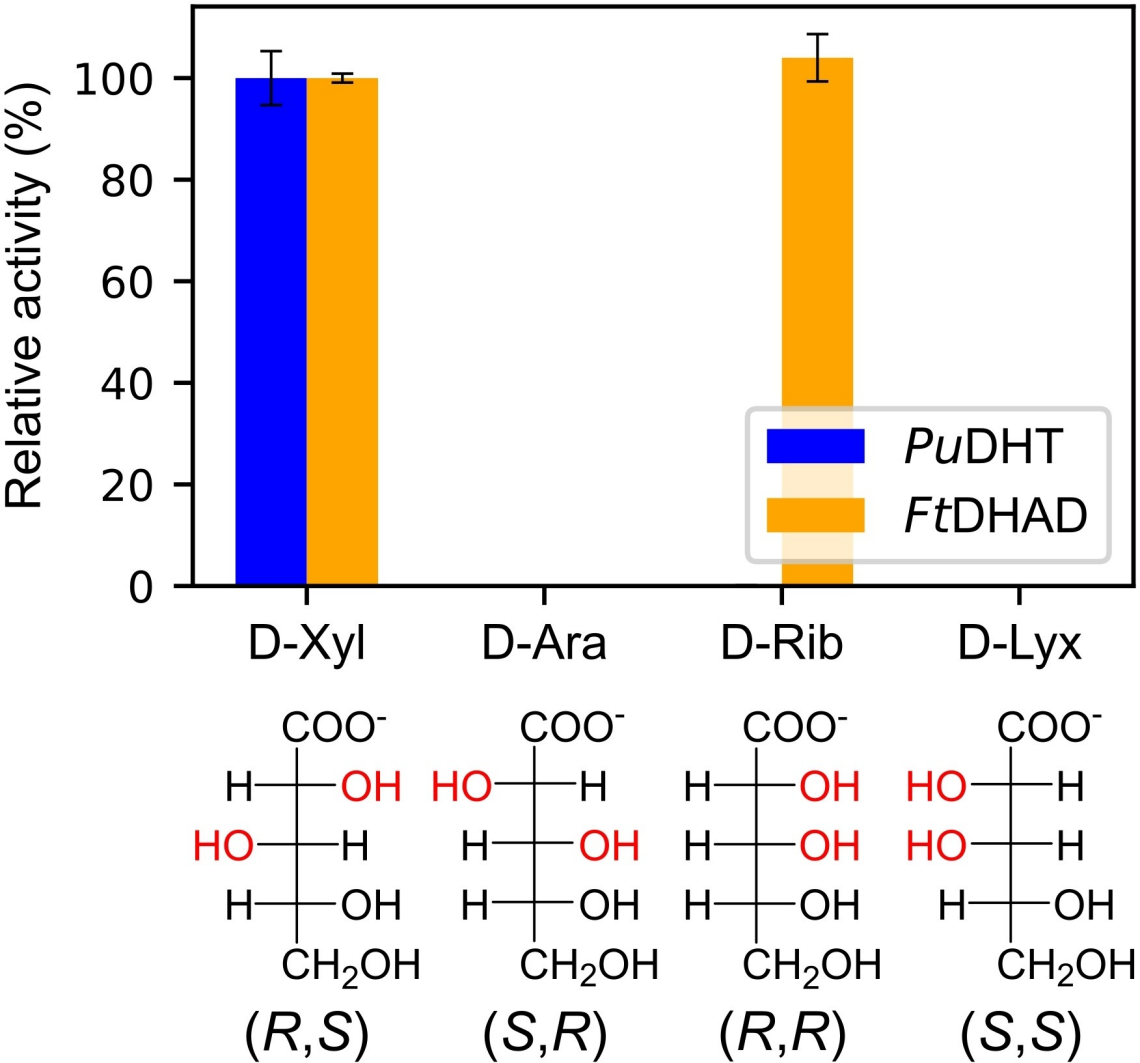
The effect of the stereochemistry at positions C2 and C3 on the substrate acceptance of SADHTs (represented by *Pu*DHT) and BCADHTs (represented by *Ft*DHAD). Exact values are provided in Table S4.

We also probed the stereoselective substrate specificity of SADHTs and BCADHTs. SADHTs only accept sugar acid substrates that have the (*R*) and (*S*) configuration at positions C2 and C3, respectively.^[8]^ Similarly, spinach DHAD (*So*DHAD), a BCADHT, has a preference for substrates with *(R)* configuration at position C2.^[24]^ Here, we examined the stereoselective substrate specificity of *Pu*DHT and *Ft*DHAD using several C5 sugar acids with different configurations in positions C2 and C3, namely D‐ribonate (*R*,*R*), D‐arabinonate (*S*,*R*), D‐xylonate (*R*,*S*) and D‐lyxonate (*S*,*S*). *Pu*DHT, similar to other SADHTs, is only active toward D‐xylonate, while the BCADHT *Ft*DHAD is equally active toward D‐ribonate and D‐xylonate (Figure 6). Molecular docking simulations using the homology models of *Pu*DHT and *Ft*DHAD demonstrate the *(R*) conformation at position C2 is essential as only in this stereoisomer both the OH group at C2 and a carboxyl oxygen at C1 are able to coordinate the catalytically essential Mg^2+^ ion. The docking simulations also indicate that D‐ribonate can be accommodated in the active site of *Ft*DHAD, maintaining its orientation and distance to Ser552, which initiates the catalytic cycle by abstracting a proton from the substrate (see above and Figure S8). However, docking simulations were not able to rationalize the stricter stereochemical requirement of SADHTs at position C3.

## Conclusions

Dihydroxyacid dehydratases, in particular those that contain a [2Fe−2S] cluster in their active sites, encompass enzymes with broad substrate spectra. Three distinct classes (SADHTs, BCADHTs and PADHTs) can be discerned based on their phylogenetic relationship (Figure 2) and substrate profiles (Table 1). We built homology models of three representative enzymes (*Pu*DHT, *Ft*DHAD and *Ss*DHAD) from each class. Together with *in silico* simulations and an analysis of sequence conservation, we were able to predict several hotspots that may play an important role in the function of these enzymes. Site‐directed mutations within these hotspots demonstrated that all of them affect the substrate selectivity and/or enzyme activity (Tables 2 and S3; Figures 4 and 5). We probed the catalytic roles of several of these hotspots in more detail. For example, the amino acid at alignment position 138 plays a role in coordinating the catalytically essential Mg^2+^ ion across all of these enzymes. Furthermore, their substrate promiscuity (or lack thereof) may be governed by three amino acids located at alignment positions 73, 224 and 261. Substituting the native proline at position 73 by a glycine (P73G) improved the substrate promiscuity of *Ft*DHAD; its P73G variant has >10‐fold improved activity toward D‐gluconate, while the activity toward DHIV (the biological substrate of the wild‐type enzyme) remains high. We used structural and sequence information to engineer dehydratases with enhanced preference for the non‐natural substrate D‐glycerate, which is an intermediate in the biotransformation of D‐glucose and glycerol to various chemicals (Scheme 1). The C‐terminal histidine was identified as relevant hotspot, as it stabilizes and orients larger sugar acid substrates in SADHTs. Replacing this histidine in *Pu*DHT by a bulkier phenylalanine indeed shifted the substrate preference of this enzyme toward shorter sugar acids, improving the activity toward D‐glycerate ∼six‐fold. In summary, the combination of *in silico* and mutagenesis studies with representatives from the three classes of the ilvD/EDD superfamily provides a “roadmap” for the engineering of optimized dehydratases for biotransformation that are of great interest to the chemical industry.

## Experimental Section

**Enzyme production and site‐directed mutagenesis**: All dehydratases in this study, except the ones from *S. solfataricus* (*Ss*DHAD) were produced using *E. coli* BL21 (DE3) in Terrific Broth (TB) and purified as described previously.^[9]^ *Ss*DHAD and its variants were produced using *E. coli* BL21 (DE3) in Autoinduction media and purified as well as activated as described previously.^[4]^ Site‐directed or saturation mutagenesis experiments were performed using either QuikChange, two‐step PCR, or overlap extension PCR. More details are described in the Supporting Information.

**Enzyme activity measurements**: Kinetic parameters of the enzymes were measured using HPLC as described previously.^[9]^ Assays with wild type and variant forms of *Pu*DHT were performed at 30 °C, while wild type and variant forms of *Ft*DHAD and *Ss*DHAD were assayed at 50 °C. More details are described in the Supporting Information.

**Molecular modelling**: Homology models were produced based on the templates with the highest sequence identity. The homology models were generated with Modeller and the resulting model with the best DOPE score was selected for further use. Molecular Mechanics parameters were generated for the [2Fe−2S] cluster and the deprotonated serine. Follow‐up MD simulations were conducted with AMBER. Molecular docking simulations were performed with AutoDock, and *in silico* mutations were performed with PyMOL using Dunbrack's rotamer library. More details are described in the Supporting Information.

**Supporting information**: Supporting experimental and computational methodology, multiple sequence alignment and structural representations of the homology models and a conversion table between sequence and alignment numbers. Activity landscape of the saturation library of alignment position 73 and C‐terminal residue, structural properties of the binding sites and substrate binding in DHAD (variants). Docked poses of D‐xylonate and D‐ribonate in DHAD. Activity data for produced DHAD (variants) and the primers and auxiliary enzymes used in this study.

Accession codes of enzymes discussed in this study (UniProtKB); *Pu*DHT: A0A4R3LQ44, *Ft*DHAD: A0A1I2J0Y3, *Ss*DHAD: Q97UB2, *Rl*ArDT: B5ZZ34, *Mt*DHAD: A0A0E8UWV6, *At*DHAD: Q9LIR4, *Cc*XylDHT: Q9A9Z2.

## Conflict of interest

The authors declare no conflict of interest.

1

## Supporting information

As a service to our authors and readers, this journal provides supporting information supplied by the authors. Such materials are peer reviewed and may be re‐organized for online delivery, but are not copy‐edited or typeset. Technical support issues arising from supporting information (other than missing files) should be addressed to the authors.

Supporting Information

## Data Availability

The data that support the findings of this study are available in the supplementary material of this article.
