# Supplementary material for: Structure‐Guided Modulation of the Catalytic Properties of [2Fe−2S]‐Dependent Dehydratases
Source: Chembiochem. 2022 Mar 23;23(10):e202200088. doi: 10.1002/cbic.202200088 (PMC9314677; doi:10.1002/cbic.202200088)
Supplement: Supplementary file 1 — Supporting Information [file CBIC-23-0-s001.pdf]

ChemBioChem

Supporting Information

Structure-Guided Modulation of the Catalytic Properties of [2Fe–2S]-Dependent Dehydratases

Okke Melse[+], Samuel Sutiono[+], Magdalena Haslbeck, Gerhard Schenk, Iris Antes[#], and Volker Sieber*

Contents

1. Methods 2
2. Multiple Sequence Alignment 6
3. Supplementary Figures 8
4. Supplementary Tables 13
5. List of primers and auxiliary enzymes used 15
6. References 17

1. Methods

Molecular Biology

Generation of mutant and production of dehydratases variants

The native sequence (WT) of *Ss*DHAD, *Ft*DHAD, and *Pu*DHT were cloned to pET28 as described in previous studies. All mutants were generated using either standard QuikChange protocol, or two-step PCR (PCR without annealing temperature), or overlap extension PCR. The list of primers is presented in **Table S7**. The plasmid containing mutant genes were sent for sequencing to confirm the mutation. The correct plasmid was then used to transform *E. coli* BL21 (DE3). Expression of all variants of *Ft*DHAD and *Pu*DHT was done as previously described. All the variants contain hexahistidine tag in the N-terminus, thus were purified using Ni NTA column and the final buffer was exchanged to 50 mM HEPES pH 8 as described previously.[1] For *Ss*DHAD WT and its respective variants, the expression was performed in autoinduction media at 25 °C as described previously.[2] Due to the instability of *Ss*DHAD in the presence of imidazole, the enzymes were heat purified in the presence of β-mercaptoethanol as described previously.[2] The final buffer was exchanged to 50 mM HEPES pH 8.

Kinetic characterization

All sugar acids were either purchased or synthesized as described previously.[1] Dihydroxy isovalerate (DHIV) was synthesized from pyruvate using two step enzymatic approach (acetolactate synthase from *Bacilus subtilis* (*Bs*ALS), ketolacid reductoisomerase from *Meiothermus ruber* (*Mt*KARI) and formate dehydrogenase from *Candida boidinii* (*Cb*FDH) for cofactor recycling). The yield and concentration of DHIV is estimated by HPLC.

All enzyme activities were measured using HPLC as described previously.[1] In short, all sugar acids and 2-keto-3-deoxy sugar acids were separated using an anion exchange column (Metrosep A Supp 16-250, Metrohm, Germany). DHIV and 2-ketoisovalerate (KIV) were separated using an ion-exclusion column (Rezex ROA-Organic Acid H+(8%), Phenomenex, Germany). Both HPLC systems were monitored using UV detector.

Final concentration of each substrate used was 25 mM. The activity was calculated by following the formation of corresponding product over time up to 24 h. *Ss*DHAD and *Ft*DHAD activities were determined at 50 °C, while *Pu*DHT was measured at 30 °C. The lowest measurable activity is 0.034 mU/mg, recorded at the lowest concentration of the standard (0.1 mM) by HPLC after a 24 h reaction with 2.5 mg/ml enzyme (maximum enzyme used).

Screening of dehydratase libraries

Expression of the dehydratases libraries (*Ft*_P73X, *Pu*_I73X, *Pu*_H653X, *Pu*DHT_N652_H653insX) were performed in 96-Deep Well Plates (DWP). In short, *E. coli* BL21 (DE3) harbouring plasmids expressing respective libraries were grown on LB-agar containing kanamycin (50 µg/ml). Single colony was inoculated in each well of 96-DWP containing autoinduction media with kanamycin (100 µg/ml). The culture was grown into saturation at 25 °C for 36 h by shaking the DWP at 1000

rpm. The culture was harvested by transferring 200 µl of cell culture to 96 U-bottom plate and centrifuging the plate at 4000 xg for 15 min. After the supernatant was decanted, the cell pellet was stored at -80 °C until further use.

The cell was lysed by transferring 200 µl of lysis buffer (50 mM HEPES pH 8, 5 mM $MgCl_2$, and lysozyme 1 mg/ml) and incubating the plate at 1000 rpm, 40 °C for 1 h. The cell debris was pelleted by centrifugation at 4000 xg for 15 min. Activity toward D-glycerate was measured using coupled assay using lactate dehydrogenase (LDH) for porcine heart (purchased from Sigma) and NADH. Activity toward L-threonate and DHIV was measured using coupled assay using the thermostable variant of *Lactococcus lactis* ketoacid decarboxylase (7M.D) and alcohol dehydrogenase from *Bacillus stearothermophilus* (*Bst*ADH) and NADH. Activity toward D-gluconate was measured by coupled assay using aldolase from *Picrophilus torridus* (*Pt*KdgA) and LDH and NADH. The oxidation of NADH was monitored at 340 nm. All coupling enzymes were used in excess. Wild type (WT) enzyme of *Ft*DHAD was used as control for *Ft*_P73X, while *Pu*DHT WT was used for (*Pu*_I73X, *Pu*_H653X, *Pu*DHT_N652_H653insX). List of auxiliary enzymes are presented in **Table S8**.

Computational Biology

Template identification and homology modelling

To identify potential templates for the homology modelling, a protein BLAST[3] search was performed against the PDB database on the sequences of the target enzymes (*Ft*DHAD, *Pu*DHT and *Ss*DHAD). This resulted in four possible templates (**Table S5**). A multiple sequence alignment (MSA) was performed with Clustal Omega[4] using these four template DHADs and the target DHADs, and visualized using ESPript.[5] From the templates within the same DHAD class (as described in the main text) as the target DHAD, the two structures with the highest sequence identity to the target DHAD were selected to serve as template during the homology modeling. Only the chains showing the active loop conformation, *i.e.* the loop containing the catalytic serine were used. For *At*DHAD, this active loop conformation was modelled inside the structure based on the structure of *Mt*DHAD using Modeller release 9.18[6] before the generation of the homology models. Since we generate homology models of the dimer, template structures with only one monomer were duplicated and manually converted into a dimer structure. The homology models were generated using Modeller; five different models were generated for each target DHAD, and the top-ranked model based on the DOPE score was selected. The binding sites of these homology models were further refined by converting the Mg^{2+} coordinating lysine to a carboxylated lysine (KCX), after which the sidechain was placed such that the Mg^{2+} ion was coordinated in an octahedral geometry. Furthermore, conserved water molecules were manually placed inside the binding site. The protonation state of the protein residues was predicted with the PROPKA3.0 software package[7,8] at pH 7.5 containing the catalytic serine in its deprotonated form, followed by a visual check. A QMEAN analysis was performed to get a quality estimation of the generated homology models.[9] Both QMEAN4 and QMEAN6 were calculated, where the first is a scoring function consisting of a combination of structural aspects, and the latter additionally contains

terms describing the agreement of the structure with sequence-based predictions of secondary structure elements and solvent-accessibility. The QMEAN calculations were performed via the Swiss-Model webserver: https://swissmodel.expasy.org/qmean/

System preparation, parameterization and simulation setup
There are no straightforward parameters for Molecular Mechanics-based simulations of the [2Fe-2S] cluster. However, because of its role in substrate binding and catalysis, proper sampling of this cluster is important. Therefore, we parameterized a bonded model of the [2Fe-2S] cluster in DHADs. We applied a similar strategy as described by Carvalho *et al.*[10], but with Fe2 having a vacant coordination site. The parameterization was performed on the *Mt*DHAD structure (PDB: 6ovt), as this structure is applied as template for two of the target DHADs, and is resolved with a high resolution.[11] The two cysteine residues coordinating Fe1 were treated equivalently during the parameterization, as well as the two sulfur ions in the [2Fe-2S] cluster. The geometry of the 'small model' generated by MCPB.py[12], consisting of the [2Fe-2S] and sidechains of the coordinating cysteine residues, was optimized at the B3LYP/6-31G(d) level of theory using Gaussian09 (revision E.01)[13], after which the bonded terms were parameterized using the Seminario[14] method on the optimized model. Charges were fitted applying the RESP procedure[15] based on a Merz-Singh-Kohlmann population analysis[16,17] on a model consisting of the [2Fe-2S] cluster and capped coordinating cysteine residues. The ChgModB scheme was applied, meaning that all charges of the backbone heavy atoms were restrained to the values from the ff14SB[18] force field. An additional improper dihedral term was added (force constant: 10 kcal·mol^{-1}, periodicity: 2, phase: 180 degrees) to the [2Fe-2S] cluster to ensure an in-plane conformation of this cluster. The final parameters are provided in **Table S6**. The nonbonded parameters of the Mg^{2+} ion were set to the values of the IOD set from Li *et al.*[19]. The substrates were parameterized as follows: Van der Waals parameters of the substrate atoms were taken from the General Amber Force Field[20] (GAFF), and the charges were retrieved from a RESP fit based on a HF/6-31G(d) geometry optimized structure of the substrates. The force field parameters for the deprotonated serine residue were retrieved via the R.E.D. server.[15,21–23] Force field parameters for the deprotonated serine which were not in the ff14SB force field were taken from GAFF.

The protonated structures (see above) were solvated in an octahedral box consisting of TIP3P[24] water applying a buffer region of 12 Å, and counterions were added to neutralize the system using the LeaP module of the Amber18/AmberTools18 software package[25]. A distance restraint between the Mg^{2+} ion and Fe2 from the [2Fe-2S] cluster with force constant 100 kcal·mol^{-1}·Å^{-1} was applied to retain a proper binding site geometry during all simulations. Energy minimization was performed with the XMIN method in Sander from the Amber18/AmberTools18 software package applying a 20.0 kcal·mol·Å^{-2} positional restraint to the protein atoms, gradually bringing the density from 0.8 g·cm^{-3} to 1.0 g·cm^{-3} with a step size of 0.02 g·cm^{-3} by adjusting the box size. At target density, a final minimization step was performed without positional restraints. The system was subsequently heated with the following procedure: 10 ps from 0-5 K, 5-10 K, and 10-20 K respectively, in a NVT ensemble, applying the Langevin thermostat[26] with a collision

frequency of 7.0 ps^{-1}, and a 3.0 $kcal\cdot mol^{-1}\cdot Å^{-2}$ positional restraints on all atoms. Subsequently, the system was further heated applying the positional restraints solely on the substrate and backbone heavy atoms in the following sequence: 50 ps 20-50 K, 100 ps 50-100 K, 100 ps 100-200 K, followed by a 200 ps equilibration at 200 K without positional restraints. Afterwards, the temperature was increased to the target temperature (300 K for *Pu*DHT and *Mt*DHAD, 323 K for *Ft*DHAD and *Ss*DHAD) in 400 ps. Finally, the system was equilibrated for 500 ps in a NPT ensemble at the target temperature, applying the Berendsen barostat[27] with a relaxation time of 1 ps and compressibility of $44\cdot 10^{-6}$ bar^{-1} to keep the pressure constant at 1 bar. Furthermore, periodic boundary conditions were applied, and the SHAKE algorithm[28] was used on all bonds involving hydrogen atoms. A nonbonded cut-off of 12 Å was used, the particle mesh Ewald method[29] was applied for long-range electrostatics, and an integration step of 1 fs was used. After the heatup, a 100-200 ns production Molecular Dynamics (MD) simulation was performed for each system using the pmemd.CUDA MD engine from Amber18, saving the velocities and coordinates of all atoms every 10 ps.

Molecular docking and in silico mutations

Docking simulations were performed with AutoDock 4.2.6[30]. A 60x60x60 points docking grid with 0.375 Å spacing, centered on the middle between the Mg^{2+} ion and Fe2 from the [2Fe-2S] cluster, was generated with AutoGrid. For each docking simulation, 150 poses were generated with a population size of 150, and a maximum of $2.5\cdot 10^{6}$ energy evaluations with AutoDock. In the top-ranked poses obtained using the AutoDock scoring function, the substrates were mostly bound in a catalytically non-competent orientation; these poses were therefore assumed to be incorrect (see detailed description in main text). Since there are no experimental structures of a dehydratase with a bound substrate, the scoring function could not be refined to improve the performance to rank the docked poses. Additionally, the presence of the highly polarized [2Fe-2S] cluster and Mg^{2+} ion is also likely to impair the performance of the scoring function in these systems. Therefore, we selected suitable docking poses based on their ability to promote the dehydratation reaction as described in the main text. In practice, the following distances were measured for every pose, and summed up to serve as docking score (in case of the substrate carboxyl oxygen, the distance to the closest oxygen was measured): the distance between a carboxyl oxygen and the hydrogen (NH) from the backbone of Thr262, the distance between a substrate carboxyl oxygen and the OH of the side chain of Thr262, the distance between a substrate carboxyl oxygen and the Mg^{2+} ion, the distance between C2-OH and the Mg^{2+} ion, the distance between C2-H and the oxygen of Ser552 and the distance between C3-OH and Fe2. Visualization and *in silico* mutagenesis was performed with PyMol[31] using Dunbrack's backbone-dependent rotamer library[32].

2. Multiple Sequence Alignment

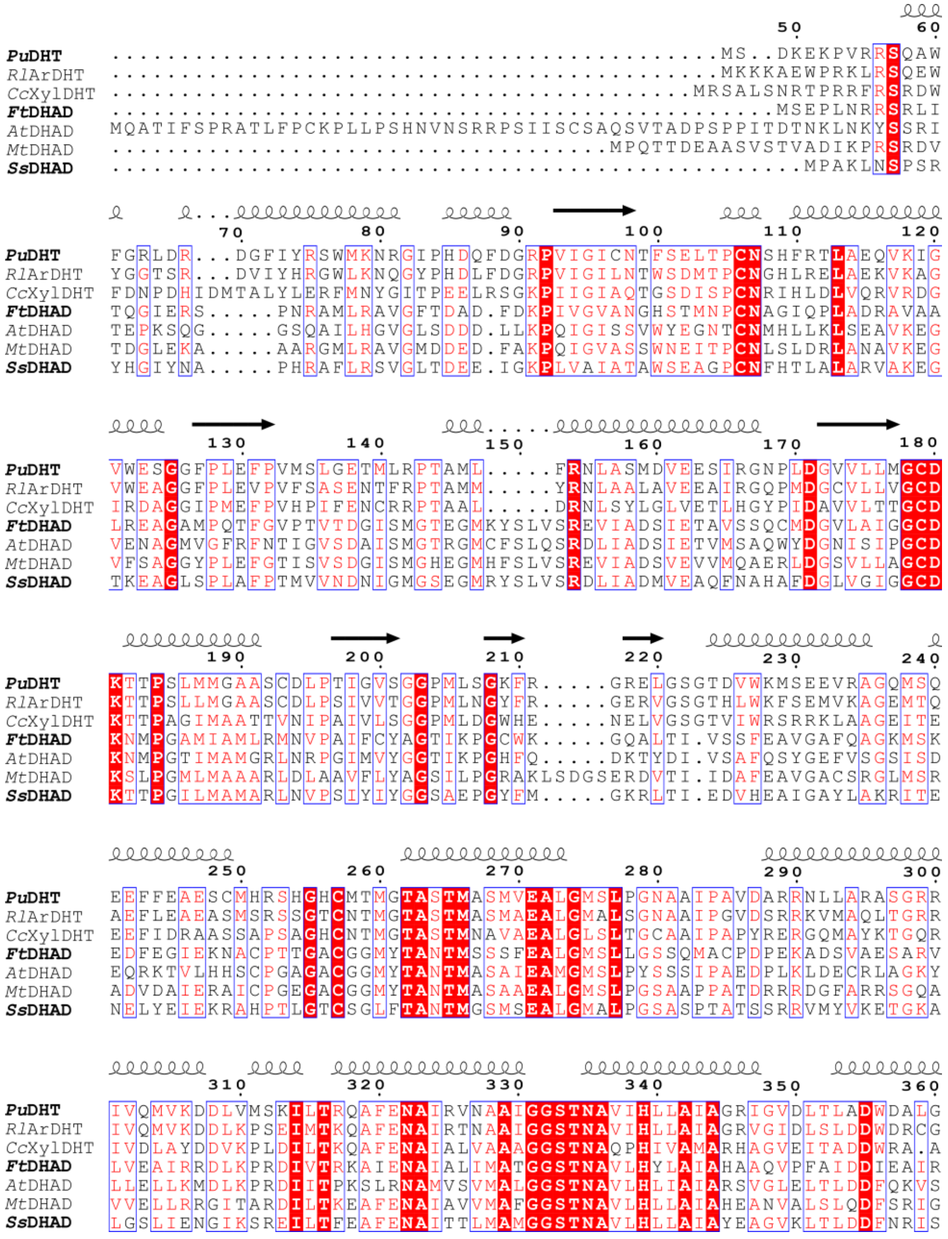

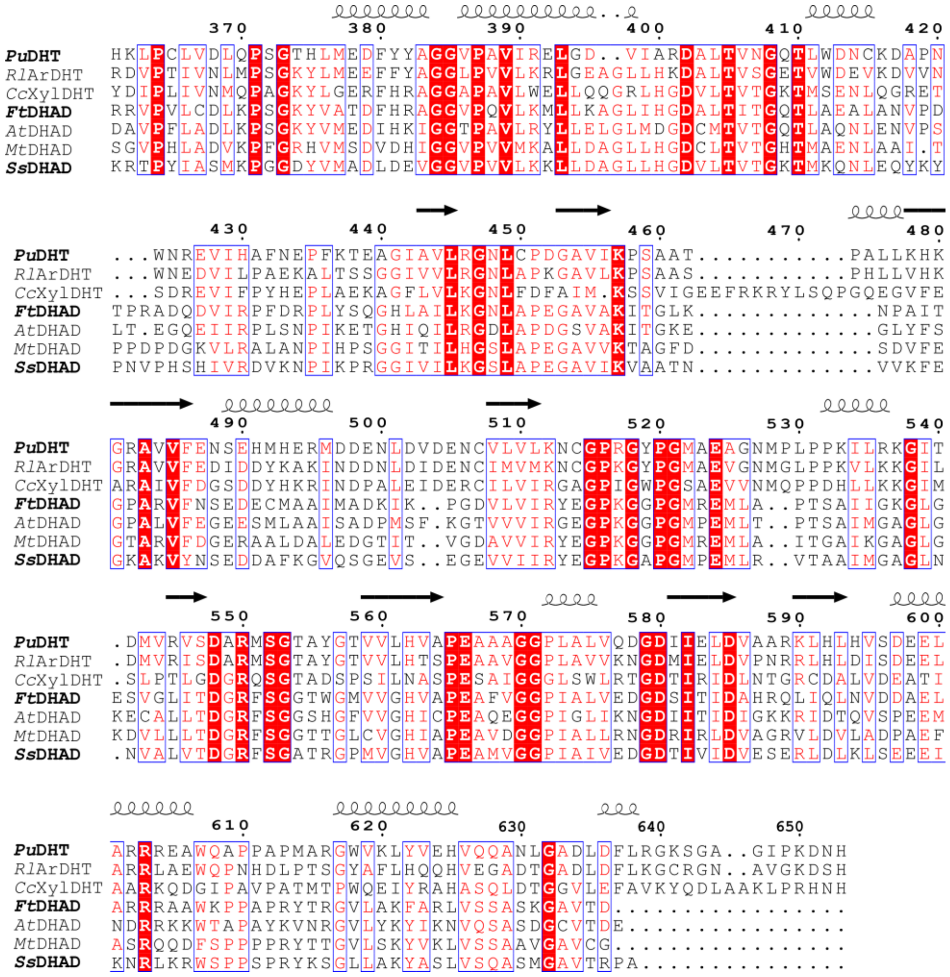

Figure S1. Full Multiple Sequence Alignment (MSA) of target- and template DHAD sequences. Enzymes in bold are the enzymes studied here. The numbers above the alignment indicate the alignment numbers, which are used throughout the manuscript. Helices and arrows above the MSA indicate the location of alpha-helices and beta-sheets, respectively, as obtained in the X-Ray structure of *Mt*DHAD (PDB-ID: 6ovt). Residues with red background indicate fully conserved residues at the respective alignment position, and residues in red font indicate a certain degree of conservation within the studied sequences. Blue boxes indicate cross-group conservation.

Table S1. Conversion between alignment numbers and protein sequence numbering

		Alignment position				
**UNIPROT ID**	**PDB-ID**	**73 (chain B)**	**224**	**261**	**552**	**138**
A0A4R3LQ44	*Pu*DHT	I24 (602)	T165	G202	S476	E89
A0A1I2J0Y3	*Ft*DHAD	P20 (582)	V164	Y201	S475	D84
Q97UB2	*Ss*DHAD	P18 (576)	E162	F199	S472	D82
B5ZZ34	5j84 -*Rl*ArDHT	Y26	T167	G204	S480	E91
A0A0E8UWV6	6ovt - *Mt*DHAD	A32	I181	Y218	S491	D96
Q9LIR4	5ze4 - *At*DHAD	G29 (604)	V173	Y210	S484	D93
Q9A9Z2	5oyn - *Cc*XylDHT	L26	T168	G205	S490	E92

3. Supplementary Figures

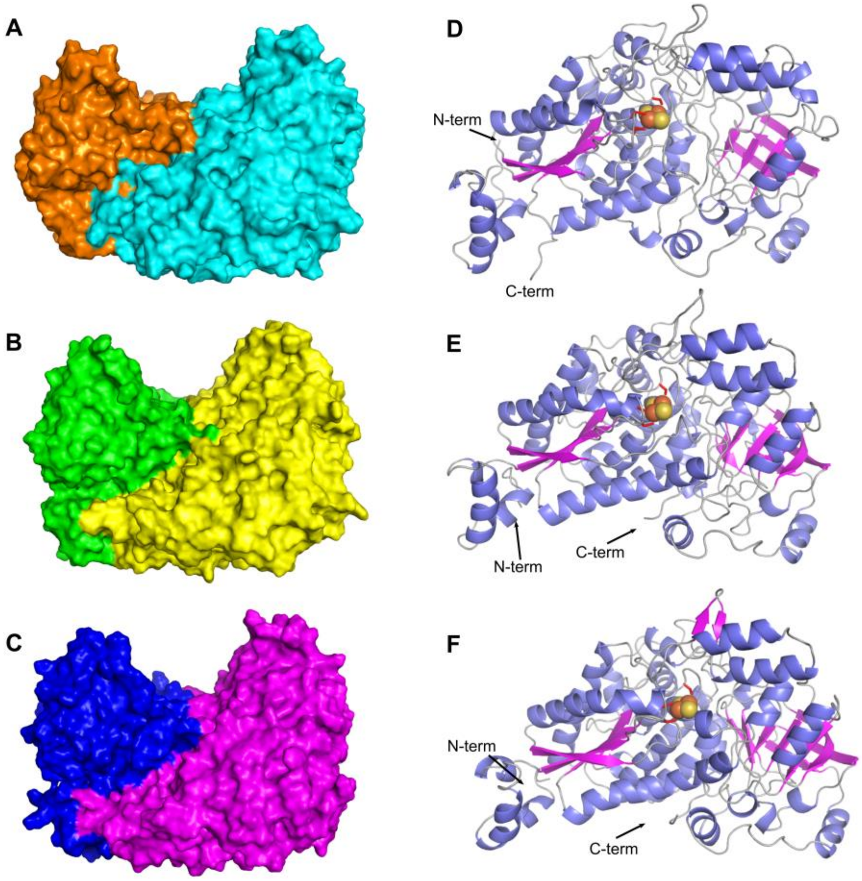

Figure S2. Structural representation of the homology models for *Pu*DHT (A,D), *Ft*DHAD (B,E) and *Ss*DHAD (C,F). The dimer is shown as surface representation (A-C) and a monomer structure is shown in cartoon representation (D-F) with the [2Fe-2S] cluster shown as spheres and its coordinating serine residues as red sticks.

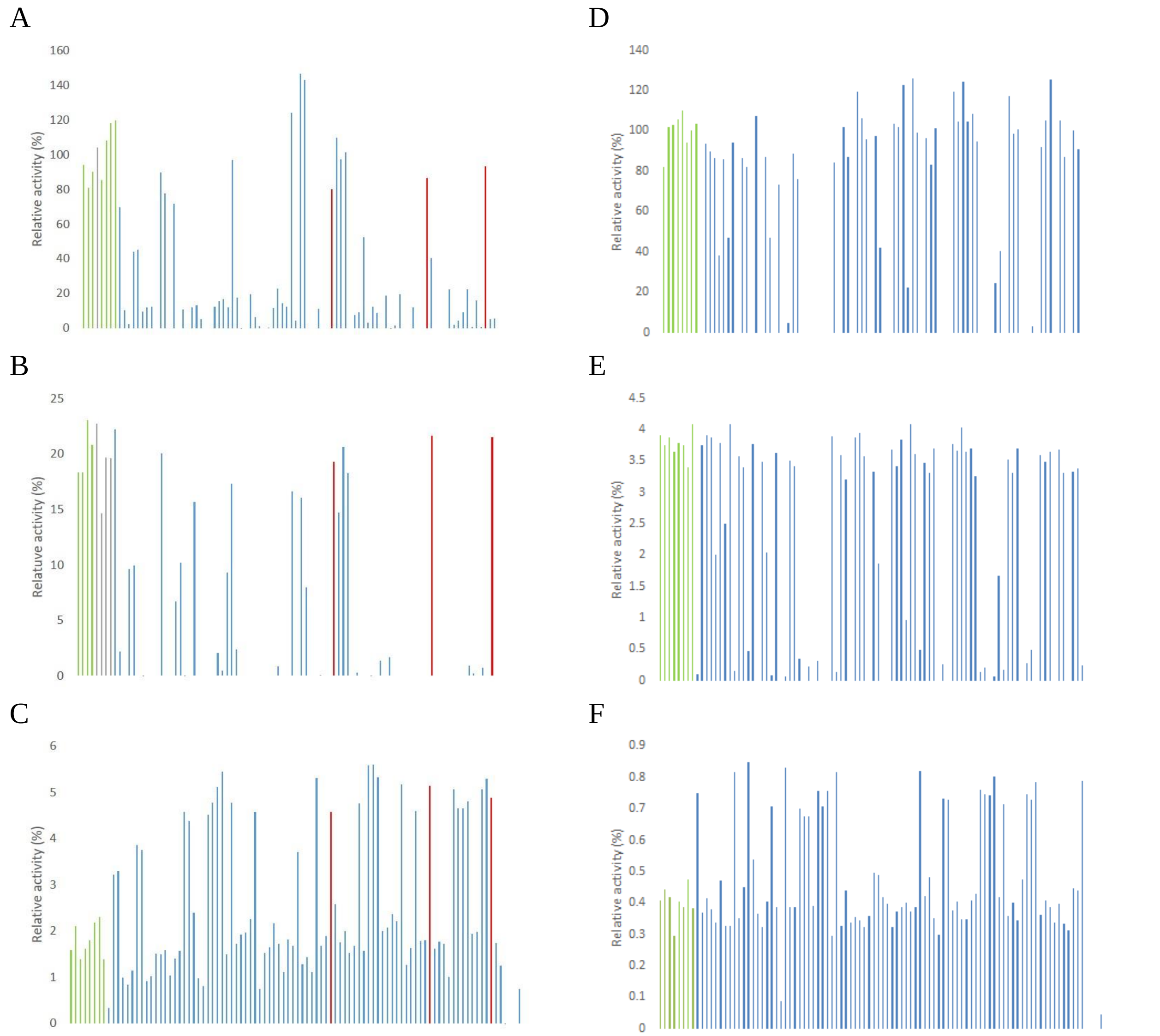

Figure S3. Activity landscape of P73X libraries for *Ft*DHAD toward DHIV (A), L-threonate (B), and D-gluconate (C) and I73X of *Pu*DHT toward D-gluconate (D), L-threonate (E), and DHIV (F). Relative activity is based on the average wild type activity (WT) of FtDHAD toward DHIV (A, B, C) or *Pu*DHT in D-gluconate (D, E, F). Green bars are the WT control of each respective enzyme. Negative controls are media only and are situated on the right. Red bars are *Ft*DHAD variants, which have P73G substitution.

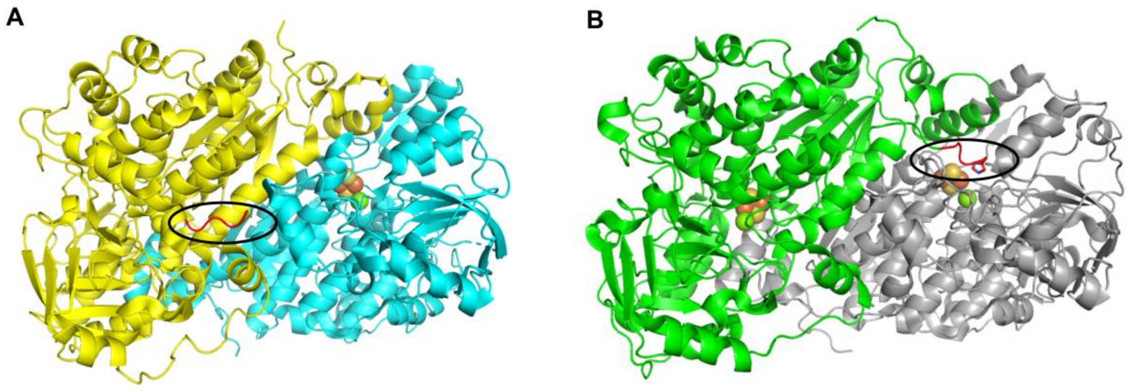

Figure S4. Location of C-terminus in BCADHTs (A) and SADHTs (B). The dimers are shown in a cartoon representation, FeS and Mg^{2+} are shown as spheres, and the C-terminus is highlighted in red. The X-Ray structure of *Mt*DHAD (PDB: 6ovt) and *Rl*ArDHT (PDB: 5j84) were used as representative for BCADHTs and SADHTs respectively.

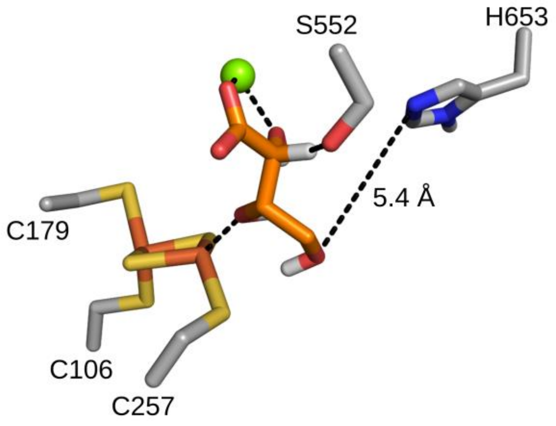

Figure S5. Docked L-threonate in the *Pu*DHT-WT, illustrating that there is no H-bond possible between the substrate and C-terminal histidine.

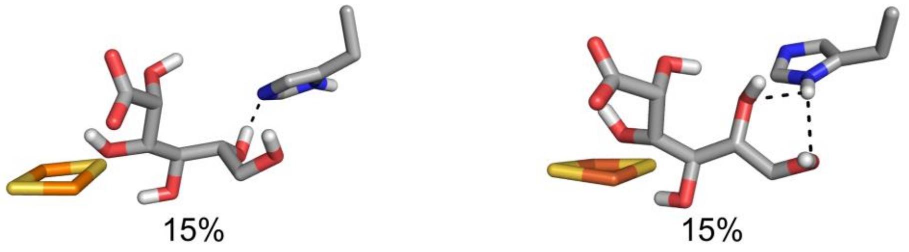

Figure S6. Remainder of observed interactions between D-gluconate and H653 during a 200 ns MD simulation of *Pu*DHT-WT. The most-sampled conformations are shown in the main text. The percentages below the binding states indicate the occurrence of the respective state during the MD simulation.

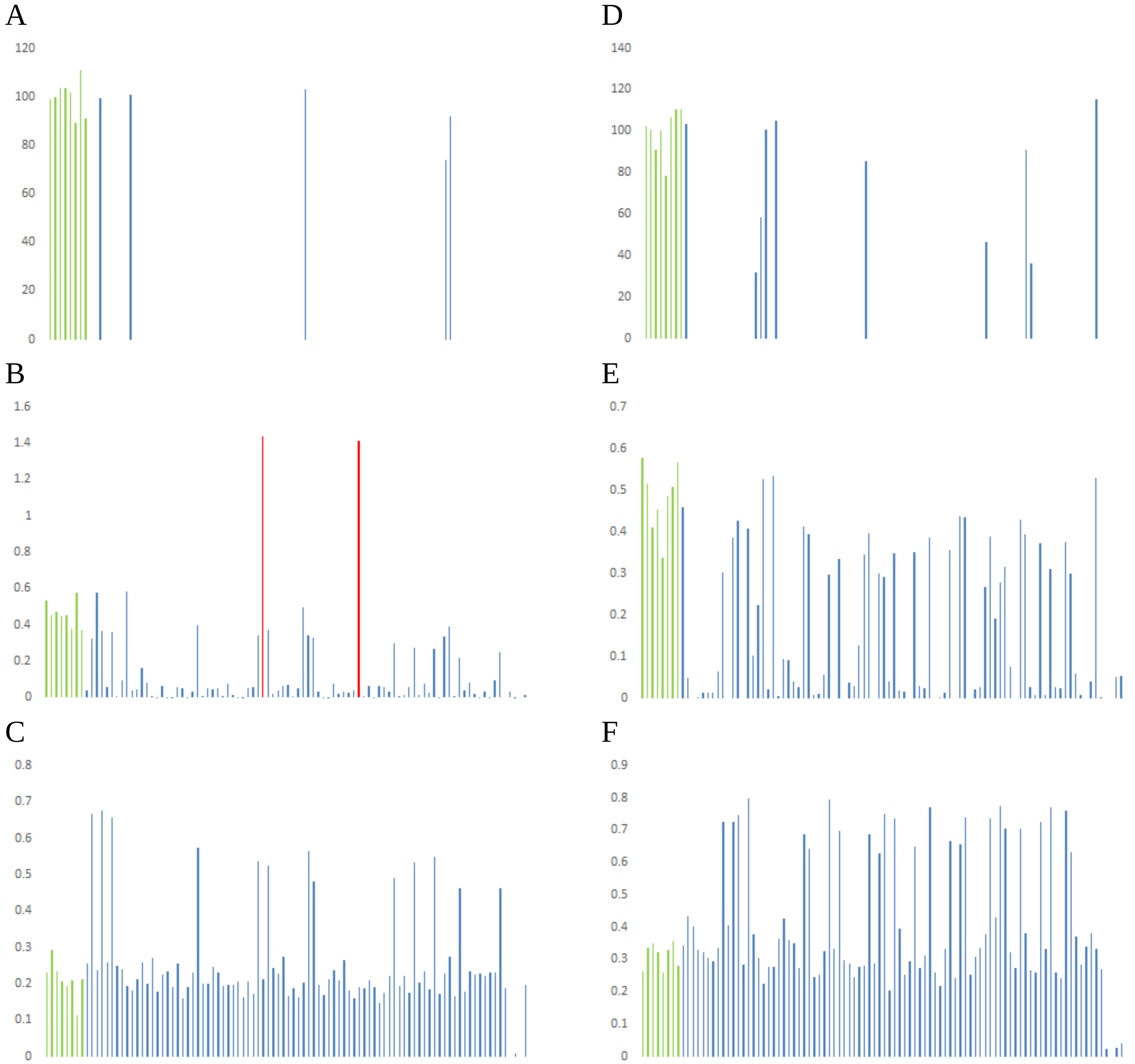

Figure S7. Activity landscape of *Pu*DHT_H653X toward D-gluconate (A), D-glycerate (B), and DHIV (C) and *Pu*DHT_N652_H653insX toward D-gluconate (D), D-glycerate (E), and DHIV (F). Relative activity is based on the average WT activity of *Pu*DHT in D-gluconate. Green bars are the WT control of each respective enzyme. Negative controls are media only and are situated on the right. Red bar are *Pu*DHT variants, which have H653F substitution.

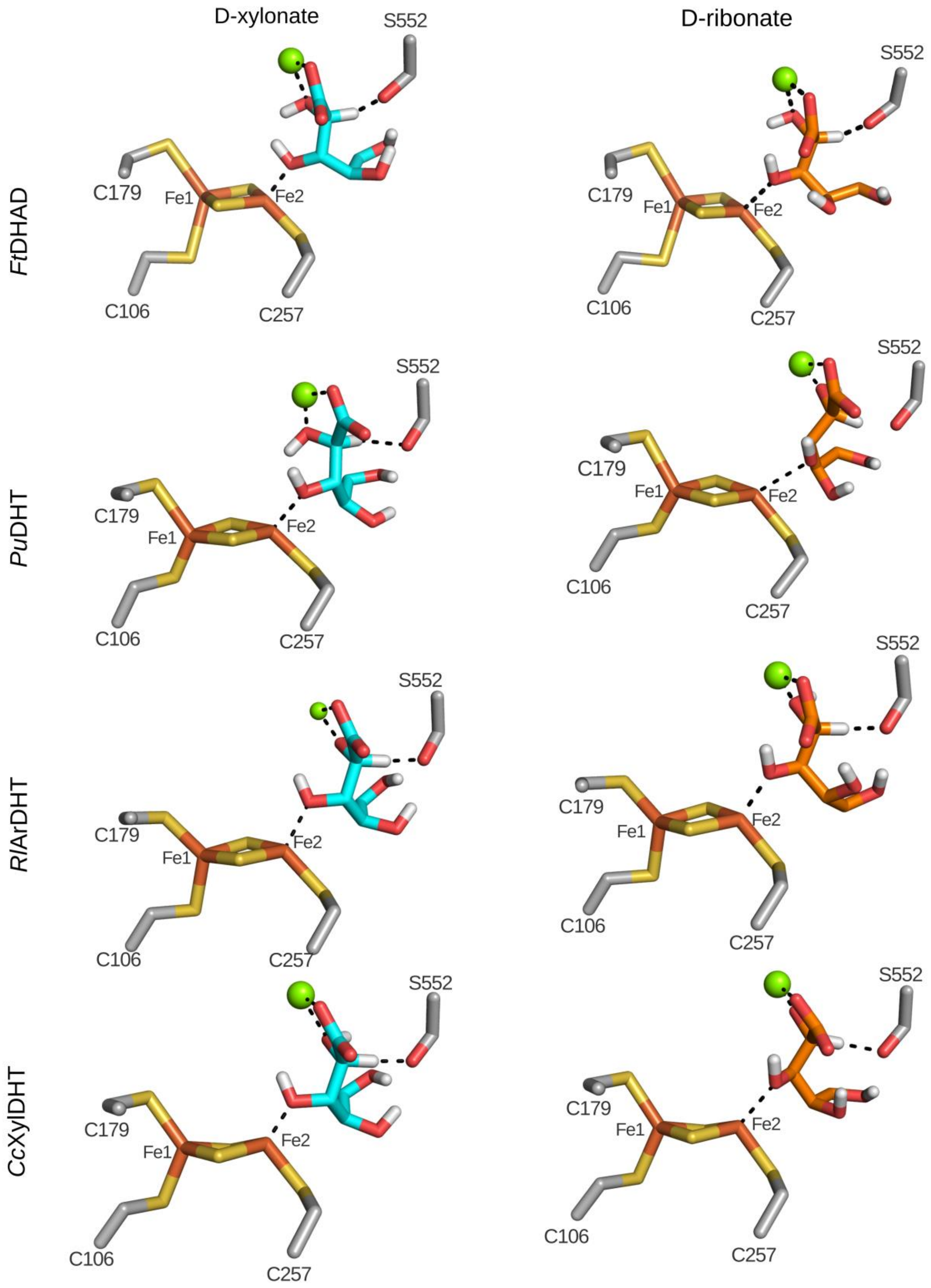

Figure S8. Docked poses of D-xylonate (left) and D-ribonate (right) in DHADs.

4. Supplementary Tables

Table S2. Quality estimation of homology models.

Target	Templates applied	QMEAN4	QMEAN6
*Pu*DHT	*Rl*ArDHT & *Cc*XylDHT	-0.62	-1.05
*Ft*DHAD	*At*DHAD & *Mt*DHAD	-1.05	-0.88
*Ss*DHAD	*At*DHAD & *Mt*DHAD	-1.34	-0.93

Table S3. Relative activity of alignment position 554/555 variants. The activity is measured relative to the wild-type. Alignment numbers are used for residue numbering.

	Rel. activity (%)
PuDHT (D-Gluconate)	
*Pu*DHT-WT (*SGTA*)	100.0 ± 3.5
*Pu*DHT-T554V (*SGVA*)	0.0 ± 0.0
*Pu*DHT-T554G (*SGGA*)	0.34 ± 0.0
*Pu*DHT-A555T (*SGTT*)	0.71 ± 0.0
*Pu*DHT-T554G, A555T (*SGGT*)	0.0 ± 0.0
FtDHAD (DHIV)	
*Ft*DHAD-WT (*SGGT*)	100.0 ± 4.9
*Ft*DHAD-G554T (*SGTT*)	0.0 ± 0.0
*Ft*DHAD-T555V (*SGGV*)	1.6 ± 0.0
*Ft*DHAD-T555A (*SGGA*)	18.4 ± 1.3
*Ft*DHAD-G554T, T555A (*SGTA*)	0.0 ± 0.0
SsDHAD (D-Xylonate)	
*Ss*DHAD-WT (*SGAT*)	100.0 ± 1.5
*Ss*DHAD-A554T (*SGTT*)	0.3 ± 0.1
*Ss*DHAD-T555V (*SGAV*)	3.1 ± 0.0
*Ss*DHAD-T555A (*SGAA*)	2.2 ± 0.0
*Ss*DHAD-A554T, T555A (*SGTA*)	0.0 ± 0.0

Table S4. Relative activity of *Pu*DHT and *Ft*DHAD in D-xylonate with different configurations of C2-OH and C3-OH.

	*Pu*DHT - WT	*Ft*DHAD - WT
D-Xyl (*R*,*S*)	100.00 ± 5.31	100.00 ± 0.88
D-Ara (*S*,*R*)	0.00 ± 0.00	0.00 ± 0.00
D-Rib (*R*,*R*)	0.09 ± 0.06	104.00 ± 4.66
D-Lyx (*S*,*S*)	0.02 ± 0.00	0.00 ± 0.00

Table S5. Sequence identity between target- and template dehydratases

	*At*DHAD (PDB: 5ze4)	*Mt*DHAD (PDB: 6ovt)	*Cc*XylDHT (PDB: 5oyn)	*Rl*ArDHT (PDB: 5j84)
*Pu*DHT	34	37	**39**	**64**
*Ft*DHAD	**49**	**51**	31	36
*Ss*DHAD	44	**51**	32	38

Table S6. Nonbonded parameters of [2Fe-2S]

Atom	Mass	LJ Radius	LJ Depth	Charge
SG1[†]	32.06	2.0000	0.2500	-0.3970
SG2[†]	32.06	2.0000	0.2500	-0.3970
SG3	32.06	2.0000	0.2500	-0.4624
S1[‡]	32.06	2.0000	0.2500	-0.4098
S2[‡]	32.06	2.0000	0.2500	-0.4098
Fe1	55.8500	1.3860	0.0136	+0.4077
Fe2	55.8500	1.3860	0.0136	+0.4991

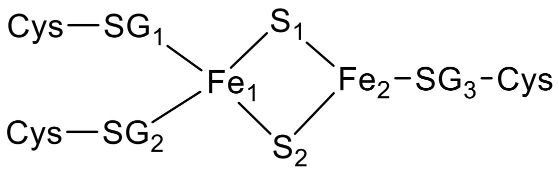

[†,‡] Both Cys1 and Cys2, and the [2Fe-2S] sulfur ions were enforced to be equal during the parameterization, both for the fitting of the bonded parameters and the RESP partial charge fitting.

5. List of primers and auxiliary enzymes used

Table S7. List of primers used in this study. The amino acid number refers to the actual position of each enzyme

Primers	Sequence (5´→ 3´)
FtDHAD_P20X.F	CGCTCANNSAATCGCGCGATGTTGCGCG
FtDHAD_P20X.R	GCGATTSNNTGAGCGCTCGATGCCTTGGGTG
FtDHAD_D84E/A.F	GTCACCGMAGGCATCTCCATGGGCACCGAGGG
FtDHAD_D84E/A.R	AGATGCCTKCGGTGACGGTCGGCACACCGAAGG
FtDHAD_V164E.F	ACCATCGAATCGTCCTTCGAGGCCGTCGGTGCG
FtDHAD_V164E.R	AGGACGATTCGATGGTCAGCGCCTGACCCTTCC
FtDHAD_Y201G.F	GGCATGGGTACCGCGAATACGATGTCCTCGTCG
FtDHAD_Y201G.R	TTCGCGGTACCCATGCCGCCGCAGGCGCCGG
FtDHAD_T478V/A.F	GGCGGAGYCTGGGGTATGGTCGTGGGACACGTC
FtDHAD_T478V/A.R	TACCCCAGRCTCCGCCGGAGAAACGGCCATCGG
FtDHAD_GT477/8TT/A.F	TCCGGCACCRCCTGGGGTATGGTCGTGGGACACG
FtDHAD_GT477/8TT/A.R	ACCCCAGGYGGTGCCGGAGAAACGGCCATCGGTG
SsDHAD_T475A/V.F	GGTGCAGYCCGTGGTCCGATGGTTGGTCATG
SsDHAD_T475A/V.R	ACCACGGRCTGCACCGCTAAAACGACCATCGG
SsDHAD_AT474/5TT/A.F	TAGCGGTACCRCCCGTGGTCCGATGGTTGGTCATG
SsDHAD_AT474/5TT/A.R	CCACGGGYGGTACCGCTAAAACGACCATCGGTAAC
PuDHT_I24X.F	GGTTTCNNSTACCGTTCGTGGATGAAGAATCGAGG
PuDHT_I24X.R	AACGGTASNNGAAACCGTCGCGGTCAAGCC
PuDHT_E89D/A.F	CTGGGCGMTACGATGTTGCGGCCTACGGCCATG
PuDHT_E89D/A.R	ACATCGTAKCGCCCAGCGACATGACCGGAAACTCG
PuDHT_T165E.F	TTCAGGCGAAGACGTCTGGAAGATGTCTGAAGAAG
PuDHT_T165E.R	AGACGTCTTCGCCTGAACCCAGTTCGCGGCC
PuDHT_G202F/Y.F	ACGATGTWTACGGCATCCACGATGGCCAGCATG
PuDHT_G202F/Y.R	ATGCCGTAWACATCGTCATGCAATGCCCATGC
PuDHT_T478G/V.F	AGCGGCGKTGCGTATGGCACGGTGGTTCTGC
PuDHT_T478G/V.R	ATACGCAMCGCCGCTCATGCGGGCGTCGGAC
PuDHT_A479T.F	GGCACCACCTATGGCACGGTGGTTCTGCATGTG
PuDHT_A479T.R	TGCCATAGGTGGTGCCGCTCATGCGGGCGTC
PuDHT_TA478/9GT.F	AGCGGCGGCACCTATGGCACGGTGGTTCTGCATG
PuDHT_TA478/9GT.R	TGCCATAGGTGCCGCCGCTCATGCGGGCGTC
PuDHT_H575X.F	GACAATNNSTAAGAATTCGAGCTCCGTCGACAAGC
PuDHT_H575X.R	AATTCTTASNNATTGTCTTTGGGTATGCCCGCGCC
PuDHT_Ins575X.F	GACAATNNSCACTAAGAATTCGAGCTCCGTCGAC
PuDHT_Ins575X.R	CTTAGTGSNNATTGTCTTTGGGTATGCCCGCGCCG
SsDHAD_D82E/A.F	TTGTGAATGMAAATATTGGCATGGGTAGCGAAGGTATGCGTTATAG
SsDHAD_D82E/A.R	AATATTTKCATTCACAACCATGGTCGGAAATGCCAGCGGAGACAG
SsDHAD_D82E.F	TTGTGAATGAGAATATTGGCATGGGTAGCGAAGGTATGCGTTATAG
SsDHAD_D82E.R	AATATTCTCATTCAAACCATGGTCGGAAATGCCAGCGGAGACAG

SsDHAD_T475V.F	GGTGCAGTCCGTGGTCCGATGGTTGGTCATG
SsDHAD_T475V.R	ACCACGGACTGCACCGCTAAAACGACCATCGG
SsDHAD_AT474/5TA.F	TAGCGGTACCGCCCGTGGTCCGATGGTTGGTATG
SsDHAD_AT474/5TA.R	CCACGGGCGGTACCGCTAAAACGACCATCGGTAAC

Table S8. List of auxiliary enzymes used in this study

Enzyme	Microorganisms	NCBI Ref. Seq.*	Substitution	Reference
*Bs*ALS	*Bacillus subtilis*	WP_003244057.1	Wild type	[33]
*Mr*KARI	*Meiothermus ruber*	WP_013014163.1	T84S	[34]
*Cb*FDH	*Candida boidinii*	O13437.1	Wild type	[35]
*Pt*KdgA	*Picrophilus torridus*	WP_048059513.1	Wild type	[36]
*Bst*ADH	*Bacillus stearothermophilus*	KFL15473.1	Wild type	[33]
*Ll*KdcA (7M.D)	*Lactococcus lactis*	PDB: 2vbf	See publication	[37]

* The NCBI reference sequences/PDB numbers presented correspond to the wild type sequences

6. References

[1] S. Sutiono, M. Teshima, B. Beer, G. Schenk, V. Sieber, *ACS Catal.* **2020**, *10*, 3110–3118.

[2] J. M. Carsten, A. Schmidt, V. Sieber, *J. Biotechnol.* **2015**, *211*, 31–41.

[3] S. F. Altschul, W. Gish, W. Miller, E. W. Myers, D. J. Lipman, *J. Mol. Biol.* **1990**, *215*, 403–410.

[4] F. Sievers, A. Wilm, D. Dineen, T. J. Gibson, K. Karplus, W. Li, R. Lopez, H. McWilliam, M. Remmert, J. Söding, et al., *Mol. Syst. Biol.* **2011**, *7*, 539.

[5] X. Robert, P. Gouet, *Nucleic Acids Res.* **2014**, *42*, 320–324.

[6] A. Šali, T. L. Blundell, *J. Mol. Biol.* **1993**, *234*, 779–815.

[7] C. R. Søndergaard, M. H. M. Olsson, M. Rostkowski, J. H. Jensen, *J. Chem. Theory Comput.* **2011**, *7*, 2284–2295.

[8] M. H. M. Olsson, C. R. Søndergaard, M. Rostkowski, J. H. Jensen, *J. Chem. Theory Comput.* **2011**, *7*, 525–537.

[9] P. Benkert, M. Biasini, T. Schwede, *Bioinformatics* **2011**, *27*, 343–350.

[10] A. T. P. Carvalho, Teixeira Ana F. S., M. J. Ramos, *J. Comput. Chem.* **2013**, *34*, 1540–1548.

[11] G. Bashiri, T. L. Grove, S. S. Hegde, T. Lagautriere, G. J. Gerfen, S. C. Almo, C. J. Squire, J. S. Blanchard, E. N. Baker, *J. Biol. Chem.* **2019**, *294*, 13158–13170.

[12] P. Li, K. M. Merz, *J. Chem. Inf. Model.* **2016**, *56*, 599–604.

[13] M. J. Frisch, G. W. Trucks, H. B. Schlegel, G. E. Scuseria, M. A. Robb, J. R. Cheeseman, G. Scalmani, V. Barone, B. Mennucci, G. A. Petersson, et al., *Gaussian 09, Revision E.01, Gaussian, Inc., Wallingford CT*, **2009**.

[14] J. M. Seminario, *Int. J. Quantum Chem.* **1996**, *60*, 1271–1277.

[15] C. I. Bayly, P. Cieplak, W. Cornell, P. A. Kollman, *J. Phys. Chem.* **1993**, *97*, 10269–10280.

[16] U. C. Singh, P. A. Kollman, *J. Comput. Chem.* **1984**, *5*, 129–145.

[17] B. H. Besler, K. M. Merz Jr., P. A. Kollman, *J. Comput. Chem.* **1990**, *11*, 431–439.

[18] J. A. Maier, C. Martinez, K. Kasavajhala, L. Wickstrom, K. E. Hauser, C. Simmerling, *J. Chem. Theory Comput.* **2015**, *11*, 3696–3713.

[19] P. Li, B. P. Roberts, D. K. Chakravorty, K. M. Merz, *J. Chem. Theory Comput.* **2013**, *9*, 2733–2748.

[20] J. Wang, R. M. Wolf, J. W. Caldwell, P. A. Kollman, D. A. Case, *J. Comput. Chem.* **2004**, *25*, 1157–1174.

[21] E. Vanquelef, S. Simon, G. Marquant, E. Garcia, G. Klimerak, J. C. Delepine, P. Cieplak, F.-Y.

Dupradeau, *Nucleic Acids Res.* **2011**, *39*, W511–W517.

[22] F.-Y. Dupradeau, A. Pigache, T. Zaffran, C. Savineau, R. Lelong, N. Grivel, D. Lelong, W. Rosanski, P. Cieplak, *Phys. Chem. Chem. Phys.* **2010**, *12*, 7821–7839.

[23] M. W. Schmidt, K. K. Baldridge, J. A. Boatz, S. T. Elbert, M. S. Gordon, J. H. Jensen, S. Koseki, N. Matsunaga, K. A. Nguyen, S. Su, et al., *J. Comput. Chem.* **1993**, *14*, 1347–1363.

[24] W. L. Jorgensen, J. Chandrasekhar, J. D. Madura, R. W. Impey, M. L. Klein, *J. Chem. Phys.* **1983**, *79*, 926–935.

[25] D. A. Case, I. Y. Ben-Shalom, S. R. Brozell, D. S. Cerutti, T. E. Cheatham III, V. W. D. Cruzeiro, T. A. Darden, R. E. Duke, D. Ghoreishi, M. K. Gilson, et al., *AMBER 18*, University Of California, San Francisco, **2018**.

[26] R. J. Loncharich, B. R. Brooks, R. W. Pastor, *Biopolymers* **1992**, *32*, 523–535.

[27] H. J. C. Berendsen, J. P. M. Postma, W. F. van Gunsteren, A. DiNola, J. R. Haak, *J. Chem. Phys.* **1984**, *81*, 3684–3690.

[28] J.-P. Ryckaert, G. Ciccotti, H. J. C. Berendsen, *J. Comput. Phys.* **1977**, *23*, 327–341.

[29] U. Essmann, L. Perera, M. L. Berkowitz, T. Darden, H. Lee, L. G. Pedersen, *J. Chem. Phys.* **1995**, *103*, 8577–8593.

[30] G. M. Morris, R. Huey, W. Lindstrom, M. F. Sanner, R. K. Belew, D. S. Goodsell, A. J. Olson, *J. Comput. Chem.* **2009**, *30*, 2785–2791.

[31] Schrodinger LLC, **n.d.**

[32] M. V. Shapovalov, R. L. Dunbrack Jr., *Structure* **2011**, *19*, 844–858.

[33] J. K. Guterl, D. Garbe, J. Carsten, F. Steffler, B. Sommer, S. Reiße, A. Philipp, M. Haack, B. Rühmann, A. Koltermann, et al., *ChemSusChem* **2012**, *5*, 2165–2172.

[34] S. Reiße, D. Garbe, T. Brück, *Biochimie* **2015**, *108*, 76–84.

[35] H. Slusarczyk, S. Felber, M. R. Kula, M. Pohl, *Eur. J. Biochem.* **2000**, *267*, 1280–1289.

[36] T. J. Gmelch, J. M. Sperl, V. Sieber, *Sci. Rep.* **2019**, *9*, 11754.

[37] S. Sutiono, J. Carsten, V. Sieber, *ChemSusChem* **2018**, *11*, 3335–3344.
